# Risk-Aware Predictive Maintenance for Aero-Engines via CVaR-Guided Reinforcement Learning and LLM-Based Risk Abstraction

**DOI:** 10.3390/s26206473

**Published:** 2026-10-13

**Authors:** Bolin Song, Yingqing Guo, Zaiyang Yu, Yao Ni, Wangyu Wu

**Affiliations:** 1 School of Power and Energy, Northwestern Polytechnical University, Xi’an 710072, China; 2021301897@mail.nwpu.edu.cn (B.S.); yqguo@nwpu.edu.cn (Y.G.); 2 Institute of Semiconductors, Chinese Academy of Sciences, Beijing 100083, China; yuzaiyang@semi.ac.cn; 3 School of Integrated Circuit Engineering, Guangdong University of Technology, Guangzhou 510006, China; 4 School of Computer Science, University of Liverpool, Liverpool L69 3DR, UK

**Keywords:** predictive maintenance, remaining useful life prediction, Conditional Value at Risk, reinforcement learning

## Abstract

A fundamental limitation of current predictive maintenance is the inability to transform prognostic uncertainty into actionable decisions, which often leads to either wasteful early interventions or catastrophic failures in aero-engines. To address this, we propose the Risk-Aware Predictive Maintenance (RAPM) framework consisting of four integrated components: a probabilistic stacked sparse autoencoder that extracts degradation features while providing distributional remaining useful life (RUL) predictions; a Monte Carlo Dropout module with Conditional Value at Risk (CVaR) that summarizes lower-tail predictive risk and produces a conservative RUL estimate for decision making; a locally deployed large language model (Qwen2.5-3B) that abstracts numerical uncertainty into discrete semantic risk levels, enabling interpretable reward shaping; and a Proximal Policy Optimization agent trained with curriculum learning to handle long-horizon sparse-reward maintenance optimization. On the NASA C-MAPSS FD001 benchmark, RAPM achieves a 96.0% optimal stopping rate with no late interventions or failure events compared with 90.2% for the strongest independent maintenance baseline. Under controlled substitution with LSTM-MCD, Transformer-MCD, CNN-MCD, and Deep Ensemble prognostic backbones, the same CVaR-LLM-PPO decision architecture maintains optimal stopping rates of 92.4–95.2% with no late interventions or failure events. Within the matched Rule-PPO ablation, CVaR increases the mean optimal stopping rate from 92.0% to 95.0% under unperturbed conditions, which is a gain of 3.0 percentage points. Under Gaussian sensor noise (σ=0.05), the gain reaches 9.0 percentage points, from 73.0% to 82.0%. These results demonstrate that the decision benefit of RAPM extends across independently trained prognostic models.

## 1. Introduction

For high-value industrial mechanical systems, maximizing operational efficiency while ensuring safe operation is essential to achieving economic returns, which is why modern industrial systems increasingly rely on intelligent predictive maintenance (PdM) strategies. In safety-critical domains such as commercial aviation, unscheduled engine shutdowns impose substantial economic costs, while prolonged continuous operation introduces significant safety risks that threaten both lives and assets [1]. Traditional time-based maintenance schedules are inherently conservative: components are replaced before failure, which provides a degree of safety assurance but sacrifices remaining useful life and cannot adapt to the heterogeneous degradation trajectories exhibited by individual engines operating under different environmental and load conditions [2]. Over the past decade, the field has undergone a transition from periodic maintenance strategies toward condition-based maintenance, and within that paradigm, from manual feature engineering through classical machine learning to deep sequence models. Convolutional neural networks (CNNs) and long short-term memory (LSTM) networks have progressively reduced mean absolute error (MAE) on the FD001 sub-dataset [3]. Building on this foundation, stacked sparse autoencoders (SSAEs) have been used to learn degradation-relevant representations for aircraft-engine RUL prediction [4], while Transformer-based architectures have pushed this frontier further [5]. Despite these advances, three fundamental limitations persist across most existing approaches.

Nearly all remaining useful life estimators report a single scalar prediction per engine cycle, implicitly assuming that the model output is a reliable proxy for the ground truth. In practice, sensor noise, model error, and distribution shift between training and deployment introduce substantial predictive uncertainty that a point estimate cannot convey [6]. Bayesian deep learning methods [7], including Monte Carlo (MC) Dropout [8] and deep ensembles [9], offer principled uncertainty decomposition but have seen limited adoption in the predictive maintenance literature, and studies that leverage the resulting distributions for downstream decision making are rarer still. Even in those studies that do quantify predictive uncertainty, it is typically treated as a secondary diagnostic—visualised post hoc as confidence intervals—rather than as a primary input to the maintenance decision.

The gap between a probabilistic RUL forecast and a concrete maintenance action is bridged informally by human experts applying domain heuristics that are seldom formalized or reproducible. An end-to-end computational framework capable of converting uncertainty into a graded, interpretable risk signal and feeding that signal directly into an optimization-based decision policy—one that can incorporate expert knowledge in a physically interpretable manner and treat probabilistic predictions as decision inputs—remains an open challenge.

Reinforcement learning (RL) has emerged as a principled framework for maintenance decision making [10], casting the problem as a Markov Decision Process (MDP) in which an agent learns to balance maintenance cost against failure risk [11,12]. However, RL agents trained purely from numerical reward signals operate as opaque function approximators: they cannot reason about the qualitative semantics of degradation stages nor adapt well to operational regimes underrepresented in the training data.

Large language models (LLMs), while demonstrating promising capabilities in industrial reasoning tasks [13], remain largely unexplored as components of RL-based maintenance control loops.

To address these three limitations simultaneously, this paper proposes a unified, end-to-end Risk-Aware Predictive Maintenance (RAPM) framework comprising four tightly coupled modules. First, a Probabilistic Stacked Sparse Autoencoder (SSAE) learns compact degradation representations through unsupervised reconstruction and performs probabilistic RUL regression via a Gaussian negative log-likelihood objective. Second, Monte Carlo Dropout with Conditional Value-at-Risk (CVaR) quantification converts the sample distribution from N=50 stochastic forward passes into a lower-tail risk summary and a conservative RUL estimate for decision making. Third, a locally deployed LLM Risk Advisor (Qwen2.5-3B-Instruct [14,15] via Ollama) generates a discrete five-level risk classification through structured reliability-assessment prompting that explicitly reasons over the dispersion gap and degradation lifecycle context [16]. Fourth, a PPO agent trained with three-phase curriculum learning incorporates the LLM risk level as a structured reward-shaping signal, learning a maintenance policy that is sensitive to predictive uncertainty while maximizing residual asset utilization within a safety-constrained optimal maintenance window.

The principal contributions of this work are as follows:

1. An integrated probabilistic degradation-to-decision pipeline: This paper presents a risk-aware predictive maintenance framework that integrates probabilistic SSAE feature extraction, CVaR-based tail risk quantification, LLM semantic risk assessment, and PPO-based maintenance optimization into a single end-to-end system requiring no human intervention.
2. CVaR as an uncertainty-aware observation signal for RL: Rather than treating predictive uncertainty as a post hoc diagnostic, we embed the CVaR estimate (α=0.05) directly into the RL state space alongside the predicted mean RUL. Ablation experiments demonstrate that this design enables the PPO agent to adopt a more risk-calibrated maintenance timing strategy.
3. LLM-enhanced reward shaping via structured prompting: We introduce a dual-stage interaction architecture in which a locally deployed 3B-parameter LLM provides cycle-level risk classifications that modulate the RL reward signal, enabling the agent to acquire engineering-semantics-aware behavior. Query throttling and semantic caching restrict fresh LLM inference to 3.1% of decision cycles, avoiding 96.9% of the queries required by naive cycle-wise invocation.
4. Three-phase curriculum learning for long-horizon maintenance policies: We design a curriculum schedule that progressively expands episode starting positions from near-failure segments through mid-life trajectories to complete lifecycle rollouts, resolving the sparse-reward challenge that causes naive PPO training on maintenance MDPs to collapse into degenerate early-stopping behavior.

The remainder of this paper is organised as follows. Section 2 reviews related work. Section 3 details the RAPM methodology. Section 4 presents and analyzes the results. Section 5 discusses limitations and future directions. Section 6 concludes.

## 2. Related Work

### 2.1. Probabilistic SSAE-Based RUL Prediction

Recent RUL research has advanced along several complementary directions. Ma et al. [4] used an SSAE with multilayer self-learning for aircraft-engine RUL prediction, while Jing et al. [17] developed a Transformer-based hierarchical latent-space VAE for interpretable RUL estimation. Domain transfer has been addressed through staged Transformer adaptation for bearing RUL prediction [18] and source-free adaptation without access to the original source data [19]. Kim et al. [20] further considered changing operating conditions through Fisher-informed continual learning. For aero-engines, recent Transformer developments include the multiscale hourglass architecture of Guo et al. [21] alongside broader surveys of data-driven RUL prediction [22].

Probabilistic prognostics has progressed in parallel through nonlinear non-Gaussian Bayesian filtering [23], physics-informed RUL models [24,25], conformal prediction intervals [26], Bayesian deep learning [7], and multi-distribution fusion [27]. The probabilistic SSAE developed in this study complements these advances by combining sparse representation learning and Gaussian RUL regression in one architecture. Its L1 penalty selects a compact latent code, while the negative log-likelihood head jointly estimates the predictive mean and aleatoric uncertainty for downstream risk-sensitive decision making.

### 2.2. CVaR Uncertainty Quantification and Risk-Sensitive Decision-Making

Embedding CVaR as an uncertainty-aware observation signal directly in the RL state bridges a gap between two largely independent lines of research: uncertainty quantification in prognostics and risk-sensitive decision making.

In prognostics uncertainty quantification, [25] proposed a physics-informed Bayesian neural network for RUL estimation in which degradation-governed differential equations regularize the posterior, leading to improved epistemic uncertainty coverage on C-MAPSS. [26] developed a conformal prediction wrapper for deep RUL models that provides distribution-free coverage guarantees without retraining. [28] extended MC Dropout to a multi-task architecture that simultaneously estimates RUL and an anomaly score, where elevated predictive uncertainty in the anomaly channel serves as an early-warning trigger. Together, these studies establish practical approaches for characterizing predictive uncertainty in RUL estimation. RAPM builds on this foundation by carrying uncertainty information forward into the maintenance state, where it directly contributes to risk-sensitive action selection.

CVaR has supported risk-sensitive policy learning in portfolio management and autonomous driving [29,30], while recent predictive-maintenance studies have increasingly connected probabilistic RUL estimation with operational decision optimization. Lee and Mitici [31] coupled CNN-based Monte Carlo Dropout prognostics with deep reinforcement learning for aircraft-engine maintenance planning. Wang et al. [32] developed a dynamic predictive-maintenance strategy based on deep-learning ensembles, and Abbas et al. [33] combined probabilistic health-state modeling with a hierarchical DRL policy on C-MAPSS. More recently, Özcan [34] integrated ensemble RUL prediction with dynamic maintenance scheduling, while Zeng and Liang [35] linked probabilistic prognostics and PPO-based optimization for group-maintenance decisions. In parallel, Zhan et al. [27] developed multi-distribution fusion for RUL uncertainty quantification, and Basora et al. [36] compared Monte Carlo Dropout, deep ensembles, heteroscedastic networks, and Bayesian inference under a common prognostic benchmark. RAPM advances this research direction by introducing lower-tail CVaR as an explicit policy input and by incorporating LLM-derived semantic risk into both state construction and reward shaping.

### 2.3. Large Language Models for Industrial Decision Support

The integration of LLMs as online risk advisors within RL training loops connects to two emerging directions: LLMs in industrial fault diagnosis and LLMs as reward shapers or planners in RL [37].

In industrial diagnostics, FaultExplainer combines PCA-based fault detection and contributing-variable analysis with LLM-generated explanations for the Tennessee Eastman Process [38]. LLMAD uses in-context examples and an anomaly-detection chain of thought to provide interpretable time-series anomaly detection [39], while FD-LLM adapts multimodal language models to fault diagnosis for complex equipment [40]. Recent work has also explored LLM-supported human–machine troubleshooting [13] and the agentic governance of preventive-maintenance policies [41]. These studies demonstrate the growing role of LLMs in industrial diagnosis and maintenance support. RAPM extends this direction by using a constrained LLM output as an online semantic risk signal that enters both the PPO state and reward-shaping process.

In the broader RL literature, LLM-assisted reward shaping has been explored in text-based environments [42] and embodied navigation tasks [43], where LLMs derive dense reward functions from natural language task descriptions. Our approach departs from this line of work in two respects. First, the LLM here operates on numerical degradation signals rather than language descriptions, which requires structured prompting that bridges the gap between sensor-derived quantities and engineering semantics. Second, the LLM output is a discrete risk level rather than a continuous reward function, reducing variance in the shaping signal and maintaining a clear separation between the LLM-derived semantic penalty and the environment’s intrinsic reward structure. Furthermore, the query throttling and semantic caching mechanisms introduced in this paper reduce the observed fresh-query rate to 3.1% of decision cycles, corresponding to a 96.9% avoided-query ratio. This directly addresses the inference cost that is seldom quantified in LLM-in-the-loop reinforcement learning.

## 3. Methodology

### 3.1. Framework Overview

The proposed Risk-Aware Predictive Maintenance (RAPM) framework is formulated as a closed-loop sensor-to-decision architecture in which raw degradation observations are progressively transformed into maintenance actions through a sequence of increasingly interpretable intermediate representations. The RAPM framework is organized into three connected modules: prognostics and uncertainty estimation, semantic risk abstraction, and risk-aware maintenance decision making. Together, these modules establish a continuous path from sensor observations to maintenance actions. This design is motivated by the observation that maintenance decisions in safety-critical systems should not depend on a point estimate alone but rather on a structured understanding of both the predicted degradation state and the reliability of that prediction.

**Figure 1:**
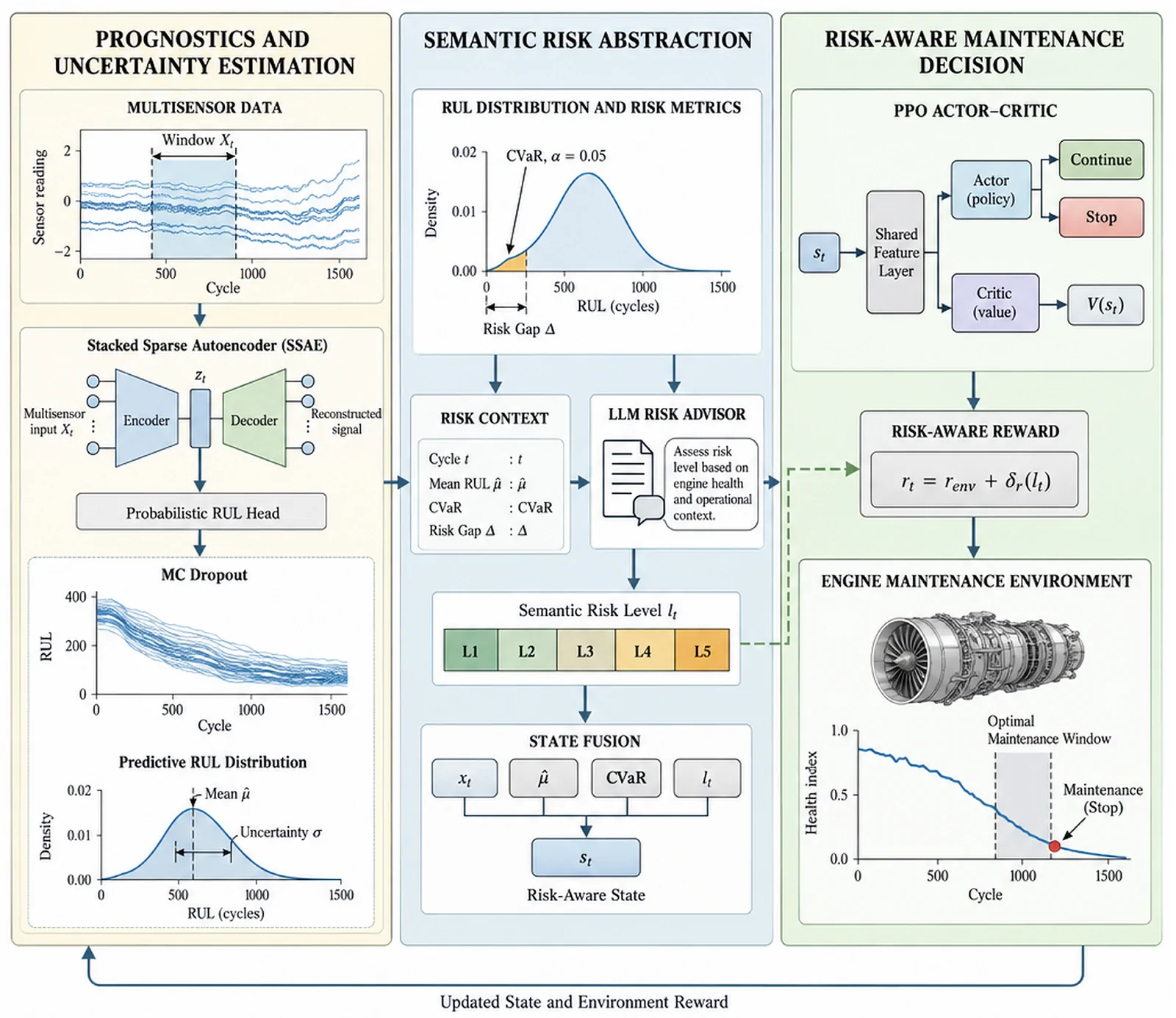
Workflow of the proposed RAPM framework.

Figure 1 presents the information flow within RAPM. At decision cycle *t*, the normalized multivariate sensor window $X_{t}$ is processed by the SSAE. The encoder extracts the latent degradation representation $z_{t}$, while the decoder reconstructs the input sequence and the probabilistic RUL head maps $z_{t}$ to the corresponding RUL estimate. Repeated stochastic forward passes with MC Dropout produce a predictive distribution characterized by its mean RUL and predictive uncertainty. CVaR at α=0.05 captures the lower tail of this distribution, while the pre-projection gap $\Delta_{t}^{\mathrm{unc}}={\hat{\mu }}_{t}-{\mathrm{CVaR}}_{0.05,t}^{G}$ isolates its dispersion component. These quantities retain both the predicted health condition and its associated risk for subsequent maintenance decision making.

The semantic risk abstraction module combines the current cycle, mean RUL, physically projected CVaR estimate, and dispersion gap into the context supplied to the LLM Risk Advisor. The resulting semantic risk level $l_{t}\in \left\{1,\ldots ,5\right\}$ is fused with the retained sensor vector $x_{f,t}$, mean RUL, and CVaR to construct the risk-aware state $s_{t}$. Based on this state, the PPO actor selects continued operation or maintenance stop, while the critic evaluates the current decision state. The same semantic risk level also enters the risk-aware reward through $\delta_{r}\left(l_{t}\right)$, integrating the LLM assessment into both state representation and policy optimization. The dashed connection in Figure 1 denotes this reward-shaping path. After the selected action is applied, the engine maintenance environment returns the updated state and reward, as indicated by the feedback path at the bottom of the figure.

A key characteristic of this architecture is that interpretability is preserved throughout the entire signal chain. At the prognostic level, the model exposes the predicted mean and uncertainty rather than a hidden latent decision score. At the risk quantification level, the CVaR estimate provides a conservative summary of the adverse tail of the predictive distribution. At the semantic level, the LLM translates numerical risk into a discrete, auditable engineering judgement. At the control level, the PPO agent outputs an explicit maintenance action under a state representation that remains physically meaningful. This layered structure is fundamentally different from end-to-end black-box policies that map sensor inputs directly to decisions without exposing intermediate diagnostic states.

From a system perspective, the framework also achieves a complete functional closure between prediction and decision. The prognostic module no longer ends with a numerical RUL estimate, and the maintenance policy is no longer trained independently of uncertainty information. Instead, the two are linked by an explicit risk interface, enabling the maintenance agent to act on what the model predicts, how uncertain that prediction is, and how that uncertainty should be interpreted in context. In qualitative terms, the RAPM framework therefore establishes a full decision-oriented pipeline that converts degradation sensing into uncertainty-aware and semantically grounded maintenance intervention, providing both operational robustness and engineering interpretability.

### 3.2. Benchmark Data Source, Generation, and Sensor Configuration

The data used in this study are from the NASA Prognostics Center of Excellence C-MAPSS turbofan engine degradation benchmark [44,45]. C-MAPSS (Commercial Modular Aero-Propulsion System Simulation) is a high-fidelity, closed-loop system-level model developed to reproduce the thermodynamic and control response of a large commercial turbofan engine over different operating and degradation states. The observations used in this work are therefore not measurements from a physical engine test rig; they are simulated sensor outputs generated by the NASA engine model. This distinction is stated explicitly because the present study provides benchmark-based methodological validation rather than physical experimental validation.

The run-to-failure trajectories are produced by prescribing initial deterioration and then progressively modifying component health parameters. For the FD001 scenario used here, degradation is associated with the high-pressure compressor (HPC), for which flow and efficiency modifiers are varied to represent progressive loss of health. C-MAPSS propagates these health changes through the coupled engine and controller model to generate the corresponding system responses. The simulation is terminated when the engine health index reaches the predefined failure boundary determined from operating margins. Random initial wear, process variability, and measurement noise are included during data generation, so different engines follow distinct degradation paths even under the same nominal operating condition [44].

Each trajectory is stored as one multivariate record per operating cycle. A record contains the engine identifier, cycle index, three operational settings, and 21 simulated sensor channels. The sensor outputs correspond to quantities normally available from an aero-engine gas-path and control system: T2, T24, T30, and T50 for total temperatures at the fan inlet, LPC outlet, HPC outlet, and LPT outlet; P2, P15, P30, and Ps30 for inlet, bypass-duct, and HPC pressures; Nf and Nc for physical fan and core speeds; NRf and NRc for corrected speeds; epr for engine pressure ratio; phi for the fuel-flow-to-Ps30 ratio; BPR for bypass ratio; farB for burner fuel-air ratio; htBleed for bleed enthalpy; $Nf_{dmd}$ and $PCNfr_{dmd}$ for demanded fan-speed quantities; and W31 and W32 for HPT and LPT coolant-bleed flows [44]. These are model-generated equivalents of measurable engine variables—not readings from named commercial sensor hardware. The present implementation uses the 14-dimensional retained sensor vector reported in Equation (9), which was obtained from the C-MAPSS channels during preprocessing. The sensor sequence is normalized before SSAE encoding, as shown in Figure 1. The 14-dimensional vector therefore denotes the model input after channel selection rather than 14 physical hardware sensors. No additional physical measurements are introduced by the RAPM pipeline; the downstream SSAE, MC Dropout, CVaR, LLM risk abstraction, and PPO modules operate only on these benchmark observations and quantities derived from them.

FD001 contains 100 training engines and 100 test engines with one sea-level operating condition and one fault mode associated with HPC degradation. Each engine begins in a nominal state with an unknown degree of initial wear and manufacturing variation. In the training set, the degradation trajectory continues until the simulated failure threshold is reached. In the test set, each trajectory is truncated before failure and NASA provides the corresponding ground-truth RUL at the final observed cycle. For training-time cycle-wise labels, the RUL is obtained from the distance between the current cycle and the end-of-life cycle of the corresponding run-to-failure trajectory.

The original NASA files are used as the sole source of the degradation trajectories; the authors do not regenerate the engine physics or alter the underlying failure process. This preserves a fixed benchmark against which different prediction and decision variants can be compared under identical lifecycle histories and makes the numerical experiments reproducible by other researchers.

This complete lifecycle structure is central to the evaluation performed in this paper. The proposed method requires not only degradation observations but also a known failure horizon in order to assess probabilistic RUL estimates, conservative tail risk, and the timing of a stop/continue maintenance policy on a common basis. Full-scale aero-engine run-to-failure tests are difficult to reproduce for algorithmic benchmarking because of safety constraints, long test duration, high cost, and limited access to proprietary fleet data. C-MAPSS therefore provides a controlled and reproducible benchmark for evaluating the methodological contribution of RAPM.

Accordingly, the results in Section 4 should be interpreted within this scope. The additional Gaussian input-noise and latent-perturbation studies are controlled stress tests of sensitivity; they are not substitutes for measurements from a physical rig. Validation using experimentally acquired or in-service aero-engine data remains an important next step for establishing deployment-level generalization.

### 3.3. Research Motivation and Methodological Design

Despite the substantial progress achieved in turbofan engine RUL prediction, most existing studies remain centered on a single objective: improving the numerical accuracy of the predicted mean. This emphasis has undoubtedly advanced prognostic modeling, yet it leaves unresolved a more consequential question for real maintenance practice: when prediction uncertainty is unavoidable, how should that uncertainty be represented, interpreted, and exploited to support better maintenance decisions. In safety-critical systems, this question is not secondary to prediction accuracy; it is often the deciding factor between prudent intervention and delayed response.

The practical difficulty lies in the mismatch between prognostic output and decision requirement. A point prediction, even when highly accurate on average, cannot distinguish between a confident estimate and an unreliable one. Two engines may receive the same predicted RUL while requiring different maintenance responses if the corresponding uncertainty differs substantially. Existing deterministic models cannot make this distinction by construction. Even probabilistic models frequently stop at reporting confidence intervals or uncertainty bands for evaluation purposes without converting uncertainty into a quantity that directly alters downstream decision behavior. As a result, prognostics and maintenance control are typically treated as adjacent but disconnected tasks [32].

This disconnect motivates the core proposition of the present work: predictive uncertainty should be elevated from a descriptive statistic to an operational decision variable [46]. To realize this proposition, the proposed framework is organized around four methodological requirements. First, degradation-sensitive features and probabilistic RUL information must be extracted within a unified prognostic model rather than through separate deterministic and post hoc uncertainty modules. Second, the predictive distribution must be compressed into a conservative risk quantity that is directly usable by a decision policy. Third, this quantitative risk must be further translated into semantic engineering language so that it can support both interpretability and policy shaping. Fourth, the resulting signals must be embedded into a maintenance control framework that remains trainable over long horizons and can exploit risk information effectively.

The design choices in RAPM follow directly from these requirements. The probabilistic SSAE addresses the first requirement by combining sparse representation learning with distributional RUL inference, thereby producing a latent health representation that is simultaneously compact, noise-suppressed, and prognostically informative. The CVaR module addresses the second requirement by transforming predictive dispersion into a conservative tail-risk measure that is better aligned with the asymmetric cost structure of maintenance decisions than conventional confidence bounds. The LLM Risk Advisor addresses the third requirement by lifting numerical uncertainty into a discrete semantic risk level, introducing an interpretable interface between probabilistic prediction and control. Finally, the PPO maintenance agent addresses the fourth requirement by integrating quantitative and semantic risk into both the observation space and the reward function, thereby closing the loop from prediction to action.

The value of this research lies precisely in this integration. The proposed framework does not treat prediction, uncertainty estimation, semantic interpretation, and decision making as isolated modules connected only superficially. Instead, it constructs an explicit signal chain in which each stage is designed to serve the next. This enables uncertainty to influence maintenance timing in a principled way rather than through manual heuristics. It also makes the overall decision process more interpretable, since the policy can be analyzed through intermediate variables that remain meaningful to engineers. In this sense, the contribution of the present work is not only algorithmic but also architectural: it redefines predictive maintenance as a unified degradation-to-decision problem rather than a sequence of loosely coupled subtasks.

More broadly, this work aims to address a growing gap as industrial AI systems transition from offline prediction benchmarks to real-world operational settings. In such contexts, predictive accuracy alone is often insufficient; instead, there is a need for frameworks that can assess the reliability of their predictions, quantify uncertainty in a cautious manner, and support decision making under that uncertainty while maintaining a degree of interpretability. The proposed RAPM framework is developed with this objective in mind. By integrating probabilistic prognostics, CVaR-based risk quantification, LLM-enabled semantic reasoning, and reinforcement-learning-based maintenance control within a unified architecture, it provides a structured approach toward more decision-aware predictive maintenance. While each component has been studied individually, their combination in this manner offers a potentially useful direction for advancing practical deployment. As such, the framework is not only methodologically meaningful but also of practical relevance for the continued development of more reliable and decision-oriented industrial AI systems.

### 3.4. MC Dropout Uncertainty Decomposition and CVaR Risk Quantification

The SSAE encoder comprises three fully connected layers with widths 128, 64, and *d*, which are followed by a decoder with a mirrored architecture. The latent dimension was selected by grid search over d∈{16,32,64}, evaluating validation-set reconstruction MSE and RUL MAE. The d=32 configuration attained the lowest validation RUL MAE and was fixed before held-out evaluation. Table 1 reports the validation results.

#### 3.4.1. Predictive Uncertainty Decomposition

At inference cycle *t*, uncertainty is estimated by performing N=50 stochastic forward passes with MC Dropout enabled, yielding predictive samples ${\left\{\left(\mu^{\left(n\right)},\log\sigma^{2(n)}\right)\right\}}_{n=1}^{N}$. The sample count was selected from a held-out sensitivity analysis over N∈{20,50,100} using the same d=32 checkpoint; Table 2 reports the resulting changes in predictive location, total dispersion, lower-tail CVaR, and interval coverage. The predictive mean is computed as

(1) $$\hat{\mu }=\frac{1}{N}\sum_{n=1}^{N}\mu^{\left(n\right)},$$

and the epistemic and aleatoric components are estimated as

(2) $${\hat{\sigma }}_{ep}^{2}=\frac{1}{N}\sum_{n=1}^{N}{\left(\mu^{\left(n\right)}-\hat{\mu }\right)}^{2},$$

(3) $${\hat{\sigma }}_{al}^{2}=\frac{1}{N}\sum_{n=1}^{N}\exp\left(\log\sigma^{2(n)}\right).$$

The total predictive variance is then defined by

(4) $${\hat{\sigma }}^{2}={\hat{\sigma }}_{ep}^{2}+{\hat{\sigma }}_{al}^{2},\;{\hat{\sigma }}^{2}\geq 10^{-6}.$$

In the above formulation, *t* denotes the current inference cycle, *N* the number of stochastic forward passes, and *n* the corresponding sample index. The quantities $\mu^{\left(n\right)}$ and $\log\sigma^{2(n)}$ represent the predicted mean RUL and log-variance from the *n*-th forward pass, respectively. The Monte Carlo estimate $\hat{\mu }$ provides the predictive mean, while ${\hat{\sigma }}_{ep}^{2}$ captures the variance induced by model uncertainty and ${\hat{\sigma }}_{al}^{2}$ reflects the data-dependent noise level. Their sum ${\hat{\sigma }}^{2}$ therefore characterizes the overall predictive dispersion with a small lower bound imposed to ensure numerical stability in subsequent computations.

Unlike prior prognostic studies that report epistemic and aleatoric uncertainty primarily for post hoc interpretation, the present framework is designed for downstream control. In this setting, the decision module does not require a separate attribution of uncertainty sources; instead, it requires a single quantity that directly reflects the reliability of the current prediction. For this reason, the two components are aggregated before risk quantification. This design removes the need for additional weighting heuristics and establishes a direct link between predictive dispersion and maintenance conservatism.

**Figure 2:**
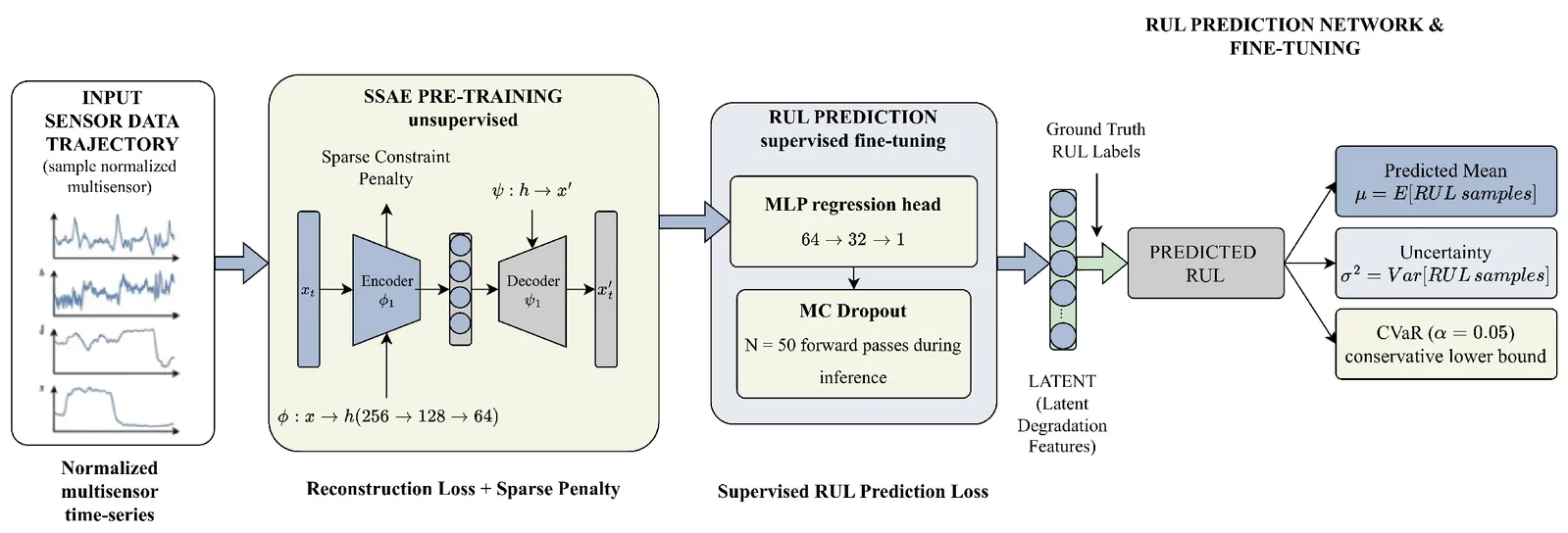
Probabilistic SSAE and CVaR estimation.

Figure 2 illustrates that uncertainty estimation is embedded within the prognostic pipeline rather than appended as an auxiliary diagnostic step. The latent degradation representation learned by the SSAE is used not only for RUL regression but also for distributional inference through stochastic sampling. This integrated design distinguishes the proposed method from deterministic feature-extraction pipelines followed by externally imposed uncertainty analysis.

#### 3.4.2. CVaR as a Decision-Oriented Risk Metric

The predictive distribution must next be transformed into a quantity suitable for maintenance decision making. Standard lower confidence bounds are often sensitive to calibration quality [47] and are therefore not ideal when the objective is operational robustness rather than interval reporting. In particular, maintenance decisions are inherently asymmetric: a missed late-stage risk is considerably more costly than a conservative early intervention.

To address this issue, the proposed framework adopts Conditional Value-at-Risk (CVaR) at confidence level α=0.05 as the uncertainty-aware risk metric. Under a moment-matched Gaussian approximation of the predictive distribution, CVaR is given by

(5) $${\mathrm{CVaR}}_{\alpha ,t}^{G}={\hat{\mu }}_{t}-{\hat{\sigma }}_{t}\frac{\phi (z_{\alpha })}{\alpha },$$

where $z_{\alpha }=\Phi^{-1}\left(\alpha \right)$ and ϕ(·) denotes the standard normal probability density function. For α=0.05, $\phi (z_{0.05})/0.05\approx 2.063$.

A confidence bound is determined by a single quantile location, whereas CVaR aggregates the full lower-tail mass below that quantile. The resulting risk signal responds jointly to predictive location and dispersion and is therefore aligned with the asymmetric cost structure of maintenance intervention.

Gaussian NLL specifies the conditional distribution associated with each stochastic forward pass, while aggregation across MC Dropout passes produces the finite Gaussian mixture

$$p_{\mathrm{MC}}\left(y_{t}|x_{t}\right)=\frac{1}{N}\sum_{n=1}^{N}N\;\left(y_{t};\mu_{t}^{\left(n\right)},\sigma_{t}^{2(n)}\right),$$

whose lower tail is not constrained to be Gaussian. The adequacy of the moment-matched representation in Equation (5) is evaluated directly on held-out engine trajectories through calibration analysis and an empirical lower-tail estimator.

For cycle *t*, the N=50 stochastic passes yield ${\left\{\mu_{t}^{\left(n\right)},\log\sigma_{t}^{2(n)}\right\}}_{n=1}^{N}$, where $\sigma_{t}^{\left(n\right)}=\exp\left[\log\sigma_{t}^{2(n)}/2\right]$. Samples incorporating both epistemic and aleatoric uncertainty are obtained as

$${\tilde{y}}_{t}^{\left(n\right)}=\mu_{t}^{\left(n\right)}+\sigma_{t}^{\left(n\right)}\epsilon_{t}^{\left(n\right)},\;\epsilon_{t}^{\left(n\right)}∼N\left(0,1\right).$$

Let ${\tilde{y}}_{t,(1)}\leq \cdots \leq {\tilde{y}}_{t,(N)}$ denote the corresponding order statistics. The empirical lower-tail CVaR is

$${\mathrm{CVaR}}_{\alpha ,t}^{\mathrm{emp}}=\frac{\sum_{i=1}^{K}{\tilde{y}}_{t,(i)}+\rho \;{\tilde{y}}_{t,(K+1)}}{\alpha N},\;K=\left\lfloor \alpha N\right\rfloor ,\;\rho =\alpha N-K.$$

For N=50 and α=0.05, K=2 and ρ=0.5. The estimator discrepancy is measured before physical clipping as

$$e_{t}^{\mathrm{CVaR}}={\mathrm{CVaR}}_{\alpha ,t}^{G}-{\mathrm{CVaR}}_{\alpha ,t}^{\mathrm{emp}}.$$

Positive values correspond to a higher Gaussian lower-tail RUL estimate. Cycle-wise skewness, excess kurtosis, and the Anderson–Darling statistic are computed from the same predictive samples. Reliability is evaluated at nominal central coverage levels from 0.10 to 0.90 in increments of 0.10 together with 0.95 coverage; confidence intervals are obtained by engine-level bootstrap resampling. The decision-sensitivity analysis applies the same physical constraint and downstream policy to both CVaR estimators.

To preserve physical consistency, the conservative estimate is constrained as

(6) $${\mathrm{CVaR}}_{t}=\max\left(0,\min\left({\mathrm{CVaR}}_{\alpha ,t}^{G},{\hat{\mu }}_{t}\right)\right),$$

The dispersion component supplied to semantic risk abstraction is defined before this physical projection:

(7) $$\Delta_{t}^{\mathrm{unc}}={\hat{\mu }}_{t}-{\mathrm{CVaR}}_{\alpha ,t}^{G}={\hat{\sigma }}_{t}\frac{\phi (z_{\alpha })}{\alpha }.$$

Here, ${\hat{\mu }}_{t}$ denotes the MC predictive mean at inference cycle *t*. At α=0.05, Equation (7) gives $\Delta_{t}^{\mathrm{unc}}=2.063{\hat{\sigma }}_{t}$, which is invariant to an additive translation of the predictive mean. The bounded ${\mathrm{CVaR}}_{t}$ retains the physically admissible lower-tail RUL, whereas $\Delta_{t}^{\mathrm{unc}}$ carries predictive dispersion without a systematic location error. Small and large values therefore denote concentrated and diffuse predictive distributions, respectively.

### 3.5. LLM Risk Advisor

#### 3.5.1. Motivation and Semantic Role

Although $\left({\hat{\mu }}_{t},{\mathrm{CVaR}}_{t}\right)$ provides a compact quantitative characterization of degradation and uncertainty, numerical quantities alone do not fully express maintenance semantics. The same conservative RUL estimate may indicate substantially different operational meaning depending on lifecycle context. As a result, directly thresholding numerical outputs often yields rigid decision rules that lack interpretability and adapt poorly across degradation stages.

To address this limitation, the proposed framework introduces a local LLM Risk Advisor that maps the current prognostic state to a discrete risk level $l_{t}\in \left\{1,2,3,4,5\right\}$. The LLM does not replace either the prognostic model or the reinforcement learning agent; rather, it acts as an intermediate semantic layer that converts numerical uncertainty into an interpretable risk representation. This role is deliberately narrow. The model is not asked to generate maintenance actions but rather to provide structured risk assessment that can be consumed by both the control policy and human operators.

This design differs from existing LLM-assisted industrial workflows in two respects. First, the LLM is embedded online within the decision pipeline rather than used offline for explanation after prediction. Second, its output space is discrete and constrained, which preserves the optimization role of PPO while enabling the maintenance policy to exploit lifecycle-aware semantic information.

#### 3.5.2. Coupling with Probabilistic RUL Estimation

The LLM is coupled to the probabilistic RUL estimator through a constrained numerical-to-semantic interface. At inference cycle *t*, the probabilistic SSAE and MC Dropout first produce the predictive RUL distribution, from which the mean RUL ${\hat{\mu }}_{t}$, the physically bounded lower-tail estimate ${\mathrm{CVaR}}_{t}$, and the dispersion gap $\Delta_{t}^{\mathrm{unc}}$ are obtained. These quantities, together with the current cycle, form the prognostic context supplied to the LLM:

$$c_{t}=\left(t,{\hat{\mu }}_{t},{\mathrm{CVaR}}_{t},\Delta_{t}^{\mathrm{unc}}\right),\;l_{t}=D\left[F_{\mathrm{LLM}}\left(P(c_{t})\right)\right]\in \left\{1,\ldots ,5\right\},$$

where P(·) serializes the numerical context into the fixed reliability-assessment prompt, $F_{\mathrm{LLM}}\left(\cdot \right)$ denotes inference by the locally deployed language model, and D(·) validates the structured response and extracts the discrete risk level $l_{t}$. The numerical RUL distribution remains the output of the probabilistic SSAE; the LLM supplies its lifecycle-dependent semantic interpretation. The resulting $l_{t}$ is appended to the PPO state in Equation (9) and modulates the continuation reward through Equations (10) and (11). This separation preserves the calibration and traceability of the numerical prognostic model while allowing the operational meaning of the RUL estimate and its lower-tail uncertainty to influence both action selection and policy learning.

#### 3.5.3. Definition and Operational Meaning of the Risk Level

The proposed risk level is an ordinal, decision-oriented abstraction of the current prognostic evidence rather than a probability of failure or a direct maintenance command. It jointly reflects the predicted mean RUL, the conservative lower-tail estimate, the dispersion gap, and the lifecycle context. The ordering imposed in the LLM prompt follows the relation

$$l_{t}↑\;\mathrm{as}\;{\hat{\mu }}_{t}↓,\;{\mathrm{CVaR}}_{t}↓,\;\Delta_{t}^{\mathrm{unc}}↑,$$

where the dispersion gap is interpreted relative to the current degradation stage. Consequently, two engine states with similar mean RUL values may receive different risk levels when their lower-tail estimates or predictive dispersions differ. The five levels and their operational meanings are defined in Table 3.

These categories describe ordered regions of operational urgency rather than fixed RUL intervals. Levels 1–3 remain distinct components of the PPO state even though they introduce no additional continuation penalty. Levels 4 and 5 activate the penalties in Equation (11), while the final stop-or-continue action remains determined by the PPO policy.

#### 3.5.4. Structured Prompting and Contextual Risk Reasoning

The LLM assigns ordinal urgency from the predictive mean, projected lower-tail RUL, pre-projection dispersion gap, and current cycle. A fixed system message defines the five risk categories and the joint interpretation of remaining life and uncertainty; a numerical user message supplies $c_{t}$. The output is a JSON object containing an integer risk_level and a brief reason. Only the validated integer enters the policy state and continuation reward.

Appendix A provides a fully specified reference interface: the literal system and user prompts, decoding options, strict JSON parser, deterministic RuleRisk fallback, and the cache–throttling sequence. Invalid or unavailable responses invoke the fallback on the same numerical context. This interface preserves the distinct roles of distributional prognostics, semantic risk assessment, and PPO action selection.

#### 3.5.5. Inference Efficiency and Deployment Feasibility

A direct integration of LLM inference into every environment step would be computationally prohibitive. The proposed framework addresses this issue by introducing two complementary mechanisms.

First, query throttling restricts LLM calls to at most once every K=25 decision steps. This is justified by the smooth temporal evolution of engine degradation, for which semantic risk levels do not require cycle-by-cycle re-evaluation. Second, semantic caching reuses previously computed risk levels for nearby values of ${\hat{\mu }}_{t}$ and ${\mathrm{CVaR}}_{t}$ by discretizing them into 5-cycle bins:

(8) $$k_{\mathrm{cache}}=\left(\left\lfloor {\hat{\mu }}_{t}/5\right\rfloor \times 5,\;\left\lfloor {\mathrm{CVaR}}_{t}/5\right\rfloor \times 5\right),$$

where $k_{\mathrm{cache}}$ denotes the discretized cache key constructed from the binned predictive mean ${\hat{\mu }}_{t}$ and the corresponding CVaR estimate, which is used to reuse previously computed semantic risk levels and reduce redundant LLM queries. These mechanisms are not implementation details alone; they are necessary for making the LLM module compatible with iterative control training. The model is deployed locally, avoiding dependence on external APIs and improving suitability for industrial scenarios with strict data-governance constraints. More importantly, the LLM remains a sparse semantic advisor rather than a dominant computational component, which preserves the practicality of the overall framework.

The semantic cache is cleared at the start of each episode, and risk levels are reused only within that episode. The query timer and previous risk level are reset simultaneously, making the first decision eligible for a fresh query.

### 3.6. PPO Maintenance Agent with Curriculum Learning

#### 3.6.1. Risk-Aware State Formulation

The maintenance decision process is formulated as a Markov Decision Process in which the PPO agent observes

(9) $$s_{t}=\left[x_{f,t},{\hat{\mu }}_{t},{\mathrm{CVaR}}_{t},l_{t}\right]\in R^{17},$$

Here, $x_{f,t}\in R^{14}$ is the retained sensor vector, ${\hat{\mu }}_{t}$ is the MC predictive mean RUL, ${\mathrm{CVaR}}_{t}$ is its physically projected lower-tail estimate, and $l_{t}$ is the semantic risk level. This notation is used throughout; ${\mathrm{RUL}}_{t}$ denotes the reference remaining life used for training rewards and evaluation.

This state formulation is substantially different from conventional maintenance RL settings based solely on raw sensor inputs or point RUL estimates. By combining raw measurements, conservative statistical risk, and LLM-derived semantic risk in a unified state, the agent can distinguish between states that appear similar under deterministic prediction but differ markedly in decision relevance. Two cycles with comparable mean RUL values may require different actions if one corresponds to a concentrated predictive distribution and the other to a wide, low-confidence one. The proposed state design makes this distinction explicit and therefore enables uncertainty-aware policy learning.

**Figure 3:**
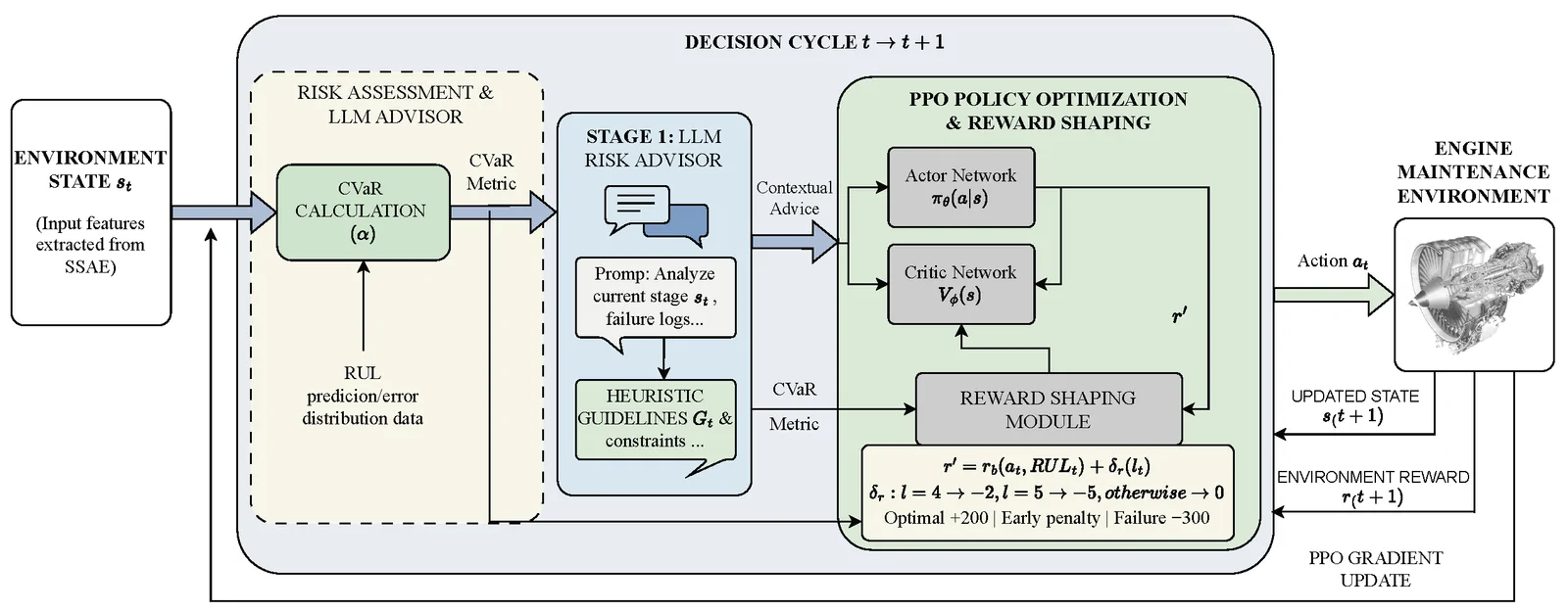
LLM-guided PPO maintenance loop.

As shown in Figure 3, the decision architecture is sequential rather than parallel. The CVaR module first converts predictive uncertainty into a conservative statistical signal; the LLM then lifts this signal into a contextual risk level; PPO finally optimizes the stop/continue policy using both representations. This layered design is one of the main methodological distinctions of the proposed framework.

#### 3.6.2. Reward Shaping with Semantic Modulation

The action space is binary, $a_{t}\in \left\{0,1\right\}$, where 0 denotes continued operation and 1 denotes maintenance stop. The reward function is defined as

(10) $$r\left(a_{t},{\mathrm{RUL}}_{t},l_{t}\right)=\left\{\begin{array}{cc} +200, & a_{t}=1,\;1<{\mathrm{RUL}}_{t}\leq W,\\ -20-1.5\cdot \max(0,{\mathrm{RUL}}_{t}-W), & a_{t}=1,\;{\mathrm{RUL}}_{t}>W,\\ -100, & a_{t}=1,\;{\mathrm{RUL}}_{t}\leq 1,\\ +1+\delta_{r}\left(l_{t}\right), & a_{t}=0,\;{\mathrm{RUL}}_{t}>0,\\ -300, & a_{t}=0,\;{\mathrm{RUL}}_{t}\leq 0, \end{array}\right.$$

with

(11) $$\delta_{r}\left(l_{t}\right)=\left\{\begin{array}{cc} -5, & l_{t}=5,\\ -2, & l_{t}=4,\\ 0, & \mathrm{otherwise}. \end{array}\right.$$

Here, $a_{t}=0$ and $a_{t}=1$ denote continued operation and maintenance stop, respectively. During PPO training, ${\mathrm{RUL}}_{t}$ is the reference remaining useful life supplied by the C-MAPSS trajectory for reward calculation, while the action is selected from the risk-aware state $s_{t}$ defined in Equation (9). The upper bound *W* specifies the maintenance planning horizon. Equation (10) assigns +200 to intervention within $1<{\mathrm{RUL}}_{t}\leq W$, whereas earlier intervention incurs a distance-dependent penalty of $-20-1.5({\mathrm{RUL}}_{t}-W)$. A stop at ${\mathrm{RUL}}_{t}\leq 1$ receives −100, and continued operation to ${\mathrm{RUL}}_{t}\leq 0$ incurs −300. For a valid continuation step, the base reward of +1 promotes useful-life utilization before semantic risk modulation. The nominal W=30 setting was fixed before held-out evaluation and places the decision window in the late-life degradation regime while retaining a multi-cycle maintenance lead time; Section 4.5 evaluates W∈{20,30,40} under both fixed-policy reclassification and matched policy retraining.

Equation (11) converts the LLM-derived risk level into an additional continuation cost. For Levels 1–3, $\delta_{r}\left(l_{t}\right)=0$, and the continuation reward remains +1. At Level 4, $\delta_{r}\left(l_{t}\right)=-2$, giving a net continuation reward of −1; at Level 5, $\delta_{r}\left(l_{t}\right)=-5$, reducing it further to −4. The incentive to continue operating therefore changes as the semantic risk rises from normal conditions to risky and critical states. Applying $\delta_{r}\left(l_{t}\right)$ to the continuation branch links the LLM assessment directly to operational urgency, while the stopping reward remains governed by the actual maintenance timing. Since $l_{t}$ is included in both the state representation and the reward function, semantic risk influences both action selection and PPO gradient updates. The same reward coefficients are used for all PPO variants evaluated in this study, providing a common decision objective for comparison.

#### 3.6.3. Curriculum Learning for Long-Horizon Decision Training

Training a maintenance policy over complete engine lifecycles is a sparse-reward problem. Under naive training, PPO frequently converges to degenerate behaviors such as premature stopping or persistent continuation, since the optimal intervention window is encountered only rarely during early exploration.

To alleviate this difficulty, a three-phase curriculum learning schedule is employed:

(12) $$f_{\mathrm{phase}}=\left\{\begin{array}{cc} 0.88, & \mathrm{Phase}\;1:\;\mathrm{steps}\;0–30,000,\\ 0.70, & \mathrm{Phase}\;2:\;\mathrm{steps}\;30,001–80,000,\\ 0.00, & \mathrm{Phase}\;3:\;\mathrm{steps}\;80,001–150,000, \end{array}\right.$$

with

(13) $$\mathrm{start\_step}=\lfloor f_{\mathrm{phase}}\cdot T\rfloor .$$

The curriculum fractions and cumulative step boundaries were fixed from the FD001 training trajectories before held-out evaluation. Their lifecycle lengths range from 128 to 362 cycles. Under the zero-based convention ${\mathrm{RUL}}_{t}=T-1-t$, $f_{\mathrm{phase}}=0.88$ retains the final 15–43 cycles and concentrates initial exploration around the intervention region; $f_{\mathrm{phase}}=0.70$ retains the final 38–108 cycles and introduces a longer pre-degradation interval; and $f_{\mathrm{phase}}=0$ exposes the policy to the complete lifecycle. The corresponding phase budgets are 30k, 50k, and 70k transitions, increasing with rollout horizon while preserving the 150k-transition total budget.

In Phase 1, episodes start close to end-of-life, allowing the agent to first learn the local stop/continue boundary under dense feedback. In Phase 2, the starting point is shifted earlier so that the agent must traverse the risk-escalation region and learn how uncertainty and semantic risk evolve prior to intervention. In Phase 3, full-lifecycle rollouts are enabled, requiring the learned policy to suppress false urgency during extended safe operation while remaining responsive near degradation onset.

The role of curriculum learning in this framework is not merely to stabilize PPO but to make the proposed risk-aware architecture trainable at realistic horizons. The upstream modules in Section 3.4 and Section 3.5 provide informative probabilistic and semantic signals, but these signals can influence control only if the policy is exposed to the relevant decision regimes with sufficient frequency during training. Curriculum learning therefore serves as the mechanism that converts the representational advantages of the upstream modules into effective long-horizon maintenance behavior.

Algorithm 1 consolidates the complete RAPM training procedure. The probabilistic SSAE is first trained on the prognostic data and then held fixed during policy optimization. At each decision cycle, its stochastic outputs are converted into a lower-tail RUL estimate, semantically interpreted by the LLM risk layer, and incorporated into the PPO state and reward.
**Algorithm 1** RAPM training with CVaR and LLM-based risk abstraction**Require:** Engine trajectories D; MC samples N=50; CVaR level α=0.05; LLM interval K=25; nominal horizon W=30; curriculum schedule ${\left\{f_{p}\right\}}_{p=1}^{3}$**Ensure:** Trained maintenance policy $\pi_{\theta }$  1:Fit the preprocessing parameters using the training engines  2:Train the probabilistic SSAE $f_{\psi }$ and freeze ψ  3:Initialize PPO actor $\pi_{\theta }$, critic $V_{\omega }$, and rollout buffer B  4:**for** p=1,2,3 **do**  5:    Set the curriculum start fraction $f_{p}$ and phase step budget  6:    **while** the phase budget is not exhausted **do**  7:        Sample an engine trajectory *e* of length $T_{e}$  8:        Initialize the episode-specific semantic cache C←⌀  9:        $t\leftarrow \lfloor f_{p}T_{e}\rfloor$, $t_{q}\leftarrow t-K$, $l_{\mathrm{prev}}\leftarrow 1$10:        $d_{t}\leftarrow \mathrm{False}$11:        **while** $d_{t}=\mathrm{False}$ **do**12:           Construct the normalized sensor window $X_{t}$ and retained vector $x_{f,t}$13:           **for** n=1,…,N **do**14:               $\left(\mu_{t}^{\left(n\right)},\log\sigma_{t}^{2(n)}\right)\leftarrow f_{\psi }\left(X_{t};\mathrm{dropout}\;\mathrm{enabled}\right)$15:           **end for**16:           Compute ${\hat{\mu }}_{t}$, ${\hat{\sigma }}_{ep,t}^{2}$, ${\hat{\sigma }}_{al,t}^{2}$, and ${\hat{\sigma }}_{t}^{2}={\hat{\sigma }}_{ep,t}^{2}+{\hat{\sigma }}_{al,t}^{2}$17:           ${\mathrm{CVaR}}_{t}\leftarrow \max\;\left(0,\min\;\left({\hat{\mu }}_{t},{\hat{\mu }}_{t}-{\hat{\sigma }}_{t}\phi \left(z_{\alpha }\right)/\alpha \right)\right)$18:           $\Delta_{t}^{\mathrm{unc}}\leftarrow {\hat{\sigma }}_{t}\phi \left(z_{\alpha }\right)/\alpha$19:           $k_{t}\leftarrow \left(5\left\lfloor {\hat{\mu }}_{t}/5\right\rfloor ,\;5\left\lfloor {\mathrm{CVaR}}_{t}/5\right\rfloor \right)$20:           **if** $k_{t}\in C$ **then**21:               $l_{t}\leftarrow C\left[k_{t}\right]$22:           **else if** $t-t_{q}\geq K$ **then**23:               $y_{t}\leftarrow F_{\mathrm{LLM}}\;\left(P(t,{\hat{\mu }}_{t},{\mathrm{CVaR}}_{t},\Delta_{t}^{\mathrm{unc}})\right)$24:               **if** $y_{t}$ satisfies the required output schema **then**25:                   $l_{t}\leftarrow D\left(y_{t}\right)$26:               **else**27:                   $l_{t}\leftarrow \mathrm{RuleRisk}\left({\hat{\mu }}_{t},{\mathrm{CVaR}}_{t},\Delta_{t}^{\mathrm{unc}}\right)$                 ▹ Reference mapping in Appendix A.328:               **end if**29:               $C\left[k_{t}\right]\leftarrow l_{t}$ and $t_{q}\leftarrow t$30:           **else**31:               $l_{t}\leftarrow l_{\mathrm{prev}}$32:           **end if**33:           $l_{\mathrm{prev}}\leftarrow l_{t}$34:           Construct $s_{t}=\left[x_{f,t},{\hat{\mu }}_{t},{\mathrm{CVaR}}_{t},l_{t}\right]$35:           Sample $a_{t}∼\pi_{\theta }\left(\cdot |s_{t}\right)$36:           Execute $a_{t}$ and calculate $r_{t}$ using Equations (10) and (11)37:           Set $d_{t}=\mathrm{True}$ if maintenance is selected or the episode terminates38:           Store $\left(s_{t},a_{t},r_{t},d_{t}\right)$ in B39:           **if** B is full or $d_{t}=\mathrm{True}$ **then**40:               Compute the advantages and returns41:               Update θ and ω using the clipped PPO objective42:               Clear B43:           **end if**44:           t←t+145:        **end while**46:    **end while**47:**end for**48:**return** $\pi_{\theta }$

The reference RUL of a training trajectory is accessed only when computing the reward in Equations (10) and (11); it is not included in the policy state. Algorithm 1 presents the logical data flow. In the implementation, the frozen SSAE outputs, CVaR values, and validated semantic risk levels for the fixed training trajectories are generated once and reused across PPO updates, preserving the same state and reward inputs without repeating the upstream inference path in every optimization epoch. During online inference, the trained policy performs the SSAE–CVaR–LLM forward path and selects an action from $s_{t}$ without access to the ground-truth RUL or PPO parameter updates.

Overall, the PPO agent should not be viewed as a conventional controller augmented with additional features. It is the final optimization layer of a structured decision chain in which probabilistic prediction, conservative tail-risk quantification, and semantic risk abstraction are explicitly coupled. This architecture is what enables the proposed framework to move beyond point-prediction-based maintenance optimization toward uncertainty-aware and interpretable decision making.

## 4. Results and Analysis

### 4.1. Experimental Protocol and Probabilistic RUL Prediction

The prognostic comparison follows the official FD001 train–test split. All models use the same retained sensor channels, 30-cycle input window, piecewise-linear RUL target capped at 125 cycles, and preprocessing statistics estimated only from the training engines. A validation subset drawn at the engine level from the training set is used for model selection, whereas the 100 truncated test trajectories are reserved for final RUL evaluation. Each prognostic model is trained independently with five random seeds, and Table 4 reports the mean and standard deviation on the official test set. The cited papers identify the model families; all numerical entries are produced by the implementations evaluated in this study under the common protocol rather than copied from the source publications. They may therefore differ from values reported under other preprocessing, data-partition, or evaluation settings.

The maintenance experiments require complete run-to-failure trajectories and are therefore evaluated separately on the 100 complete FD001 training engines using five-fold out-of-fold evaluation. In each fold, 60 engines are used for training, 20 for validation, and 20 for held-out testing, so every engine appears exactly once in a held-out fold. Preprocessing statistics and maintenance thresholds are fitted using only the corresponding training and validation engines. Each maintenance policy is retrained for its prognostic backbone and repeated with five random seeds; decision results are reported as the mean and standard deviation across seeds after combining the held-out folds.

The CVaR Rule-PPO and No-CVaR policies were compared using paired outcomes for the same engines and seeds. For each condition, the optimal-stopping difference was defined as $\Delta_{\mathrm{opt}}=100\left({\bar{I}}_{\mathrm{CVaR}}-{\bar{I}}_{\mathrm{No}-\mathrm{CVaR}}\right)$ percentage points, where *I* indicates a stop within 1<RUL≤30. Two-way paired bootstrap inference [48] used 10,000 replicates: 100 engine indices and five seed indices were sampled independently with replacement, and their Cartesian product was evaluated using identical indices for both policies. The 2.5th and 97.5th percentiles of the resampled differences define the condition-specific 95% confidence interval.

Terminal safety margins were quantified by $q_{<5}$, the percentage of evaluations ending with fewer than five cycles of true remaining life. Terminal RUL, $R_{e,s}^{\mathrm{term}}$, equals the true RUL at intervention and is set to zero for a failure. For E=100 engines and S=5 seeds,

$$q_{<5}=\frac{100}{ES}\sum_{e=1}^{E}\sum_{s=1}^{S}1\;\left(R_{e,s}^{\mathrm{term}}<5\right).$$

Two matched controls were added for the reward horizon and training schedule. For stopping-window sensitivity, the nominal W=30 RAPM policy was first frozen and its actions were reclassified under W∈{20,30,40}; PPO was then retrained separately with W=20 and W=40, while the prognostic sequences, CVaR level, LLM risk abstraction, network architecture, hyperparameters, transition budget, folds, and random seeds were held fixed. For the curriculum ablation, every episode began at t=0 throughout the same 150,000-transition budget. All remaining state, reward, model, and evaluation settings matched the three-phase RAPM configuration.

The systematic location error was separated from predictive dispersion by validation-derived additive calibration. With the residual defined as $e_{e,t}={\hat{\mu }}_{e,t}-y_{e,t}$, the coefficient for fold *f* and seed *s* was estimated by engine-balanced averaging over the corresponding validation engines $V_{f}$:

$${\hat{b}}_{f,s}=\frac{1}{|V_{f}|}\sum_{e\in V_{f}}\frac{1}{T_{e}}\sum_{t=1}^{T_{e}}e_{e,t}.$$

The coefficient was frozen before held-out evaluation, and every stochastic predictive mean was translated as $\mu_{t,\mathrm{bc}}^{\left(n\right)}=\mu_{t}^{\left(n\right)}-{\hat{b}}_{f,s}$ while both uncertainty components were retained unchanged. The same validation-only operation was applied to the official test-set prognostic evaluation using its corresponding seed-specific validation partitions. Across folds and seeds, the estimated validation residual was −16.9±0.3 cycles. Corrected RUL and CVaR trajectories were used to reconstruct the risk inputs, and the matched Rule-PPO policies were retrained with the original architecture, reward, curriculum, training budget, and random seeds. No held-out RUL label entered coefficient estimation or policy training.

To isolate the effect of the prognostic backbone, two controlled variants were constructed by replacing the SSAE with an LSTM and a Transformer, respectively. Both alternative backbones used the same two-output heteroscedastic Gaussian head to estimate the predictive mean and log-variance and were trained with the same Gaussian negative log-likelihood objective. The data partition, retained sensor channels, 30-cycle input window, RUL target, and preprocessing procedure were held unchanged. At inference, dropout remained active for N=50 stochastic forward passes [49], and the predictive mean and total variance were calculated using the same uncertainty decomposition as for the probabilistic SSAE. The resulting distributions were passed without modification to the CVaR and LLM risk-abstraction modules. A separate PPO policy was trained for each backbone because the prognostic input distribution changes after backbone replacement; however, the PPO architecture, state definition, reward function, curriculum schedule, hyperparameters, training budget, and random seeds were identical in all cases. The controlled comparison therefore changes the prognostic backbone while preserving the complete downstream decision architecture.

**Table 4:** RUL prediction performance on the official FD001 test set (mean ± standard deviation over five seeds).

Method	MAE	RMSE	NASA Score	NLL	CRPS (Cycles)	95% PICP (%)
LSTM [50]	16.8±0.4	19.6±0.5	512.4±18.2	–	–	–
1D-CNN [3]	18.1±0.5	21.0±0.6	604.7±21.5	–	–	–
Transformer [5]	14.6±0.3	17.9±0.4	438.9±15.7	–	–	–
LSTM-MCD [49,50]	17.5±0.4	20.5±0.5	540.3±19.8	0.44±0.02	19.8±0.7	90.2±1.2
Transformer-MCD [5,49]	15.8±0.4	18.9±0.5	480.2±20.1	0.41±0.02	18.5±0.6	91.0±1.1
CNN-MCD [31]	17.4±0.4	20.3±0.5	548.2±19.3	0.46±0.02	19.4±0.7	89.8±1.2
Deep Ensemble [36]	16.5±0.3	19.4±0.4	501.6±17.8	0.42±0.02	18.2±0.6	91.5±1.1
Probabilistic SSAE (ours)	19.9±0.5	24.1±0.6	978.8±24.6	0.39±0.02	17.8±0.6	92.6±1.0

NLL is evaluated on the normalized RUL target scale used to train the probabilistic output heads. MAE, RMSE, and CRPS are reported after inverse transformation to the original cycle scale, while the NASA Score is dimensionless. The same transformations and metric implementations are used for all probabilistic backbones.

Table 4 compares eight independently trained prognostic models under a common data partition and preprocessing protocol. The deterministic Transformer obtains the lowest global point-prediction errors, while Transformer-MCD remains the most accurate probabilistic model in terms of global MAE and RMSE. The probabilistic SSAE exhibits a different performance profile: although its global MAE is higher, it obtains the lowest NLL and CRPS and the 95% PICP closest to the nominal coverage level among the probabilistic models. The MAEs within the decision-critical region (RUL≤30) are 13.1, 13.8, 14.5, 14.9, and 15.6 cycles for the SSAE, Transformer-MCD, LSTM-MCD, Deep Ensemble, and CNN-MCD, respectively. Consequently, the 4.1-cycle global MAE advantage of Transformer-MCD over the SSAE does not persist near the intervention region; the SSAE instead achieves a 0.7-cycle lower MAE. This distinction between global point accuracy, probabilistic quality, and late-life accuracy motivates the controlled decision-level comparison in Section 4.2.

**Figure 4:**
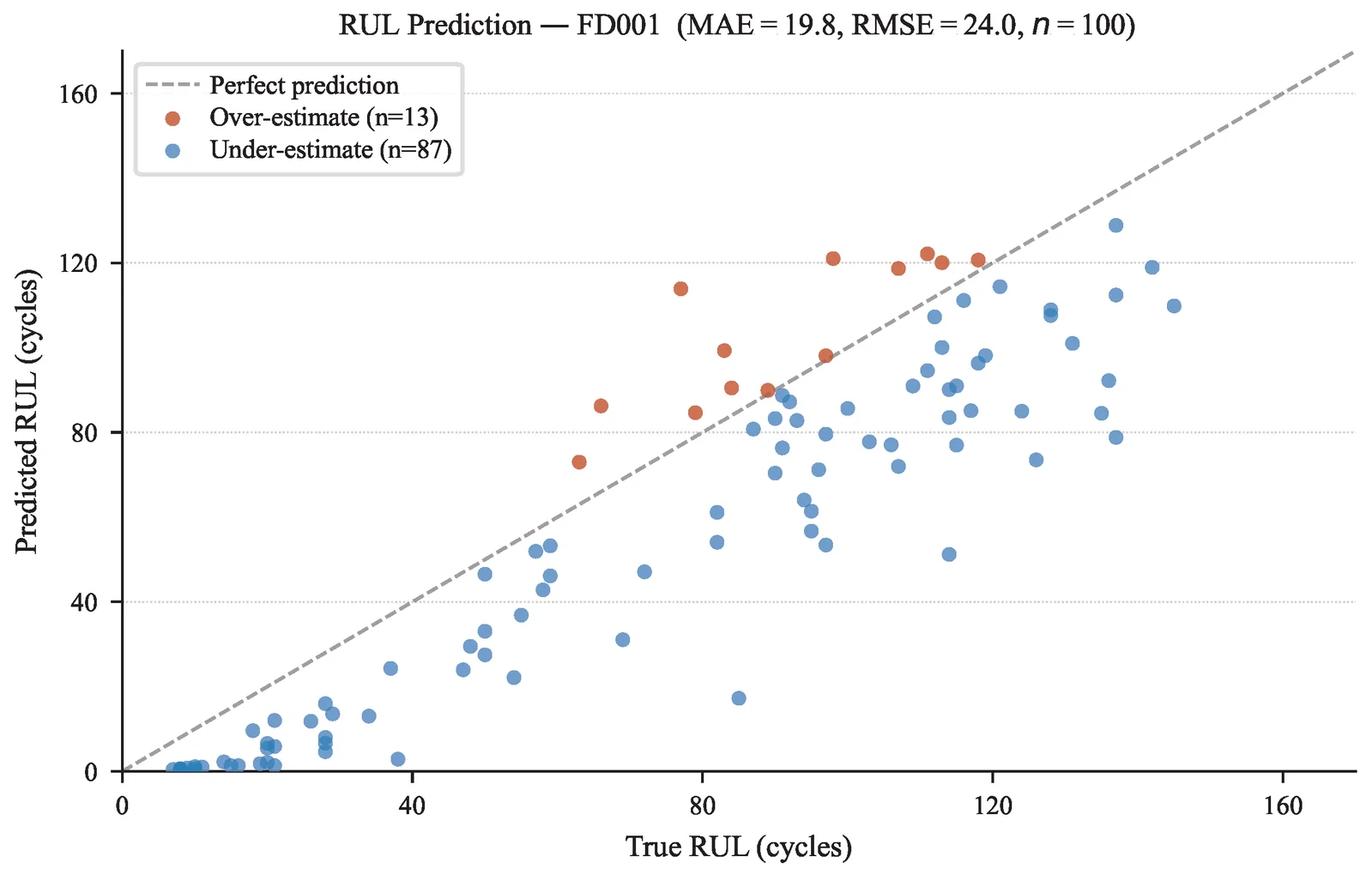
Predicted versus true RUL for a representative SSAE run on the 100 FD001 test engines (MAE 19.8 cycles; RMSE 24.0 cycles). Table 4 reports the five-seed statistics.

**Figure 5:**
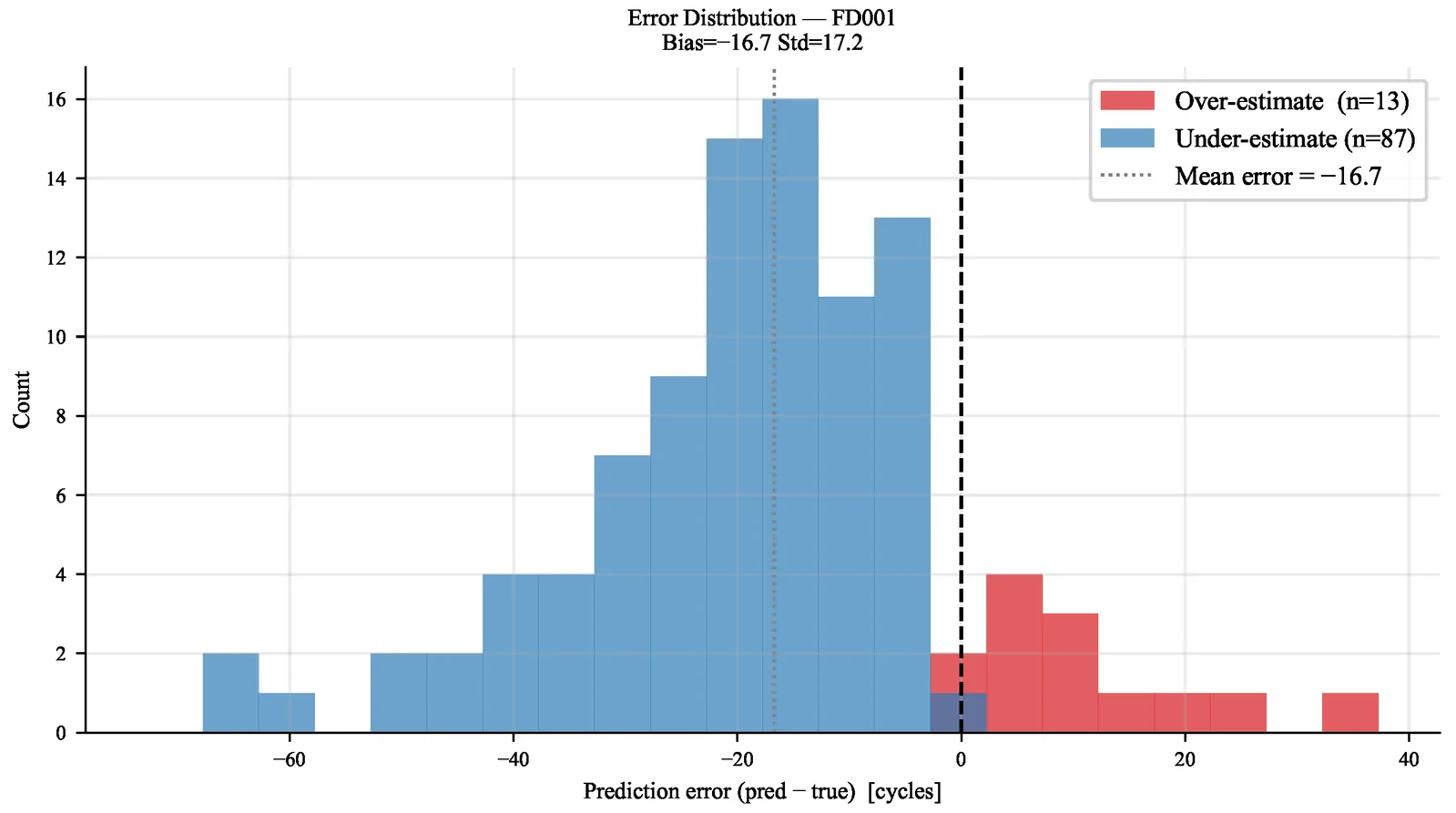
Prediction errors for the same representative SSAE run as Figure 4; residuals are predicted minus true RUL.

Figure 4 and Figure 5 expose the error structure of a representative SSAE run that is not represented by the seed-averaged scores alone. The plotted run yields an MAE of 19.8 cycles and an RMSE of 24.0 cycles; the corresponding five-seed statistics in Table 4 are 19.9±0.5 and 24.1±0.6 cycles, respectively. Figure-specific values describe the displayed run, whereas the comparative analysis uses the five-seed means. With the residual defined as predicted minus true RUL, 87 of the 100 estimates fall below the identity line, and the largest concentration lies between −30 and 0 cycles. The residual distribution is therefore dominated by underestimation rather than centered symmetrically about zero. This asymmetry has a direct decision consequence: optimistic RUL estimates can delay maintenance, whereas underestimation moves the inferred intervention point forward. Because the SSAE retains predictive dispersion, CVaR can distinguish mild conservatism from a genuinely heavy lower tail.

**Figure 6:**
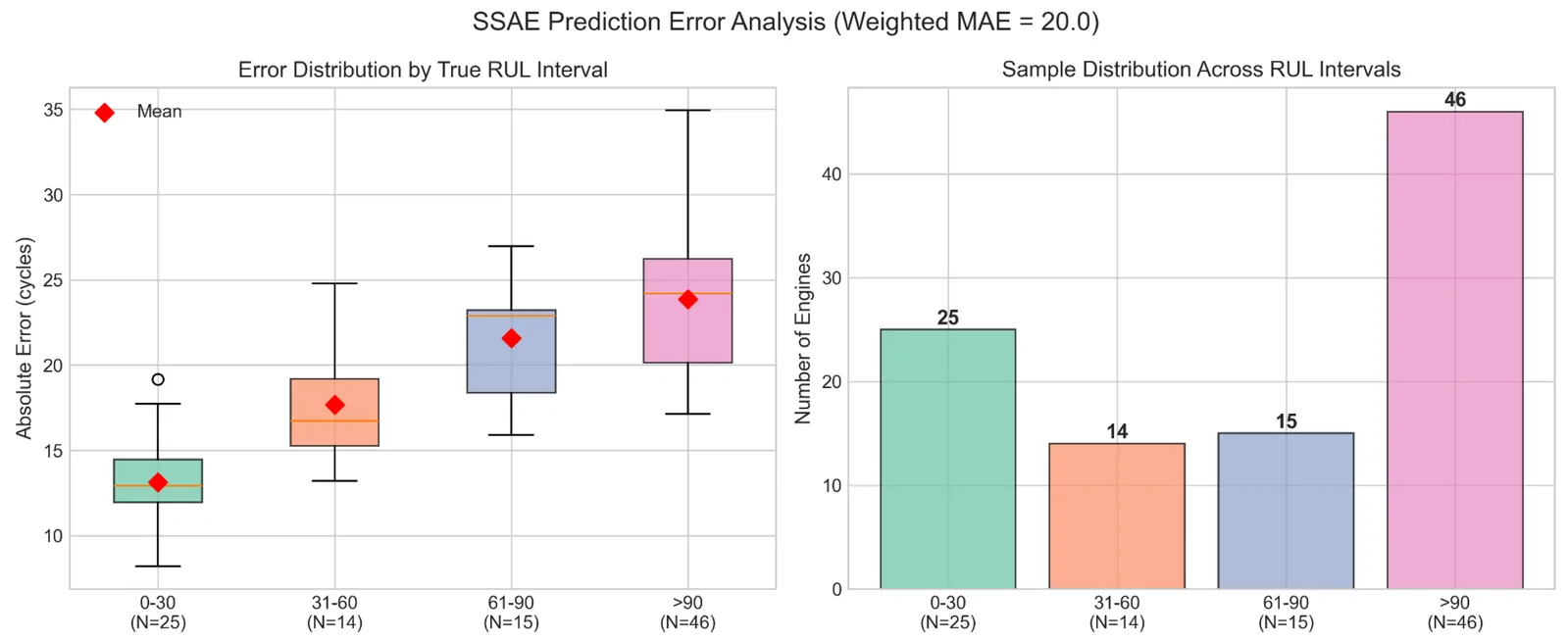
Stage-wise SSAE prediction errors and FD001 test-engine counts.

Stratification by true RUL in Figure 6 reveals a clear dependence of the error distribution on degradation stage. The mean absolute errors for the 0–30, 31–60, 61–90, and >90-cycle intervals are 13.1, 17.7, 21.5, and 24.0 cycles, respectively. The 0–30-cycle group also exhibits the narrowest interquartile range, indicating that the gain in late-life accuracy is not driven by only a small subset of engines. This progression is consistent with increasing degradation identifiability as failure approaches: late-life sensor trajectories contain stronger condition signatures, whereas high-RUL states are more similar across engines and are compressed by the 125-cycle target cap. The four intervals contain 25, 14, 15, and 46 engines, so the global error is weighted most heavily by the least-degraded group. Even under this sample composition, the weighted MAE of 20.0 cycles agrees closely with the five-seed result of 19.9±0.5 cycles in Table 4. The conditional results are more informative for maintenance than the global average alone because the smallest error and dispersion occur in the 0–30-cycle interval used to define the intervention window.

**Figure 7:**
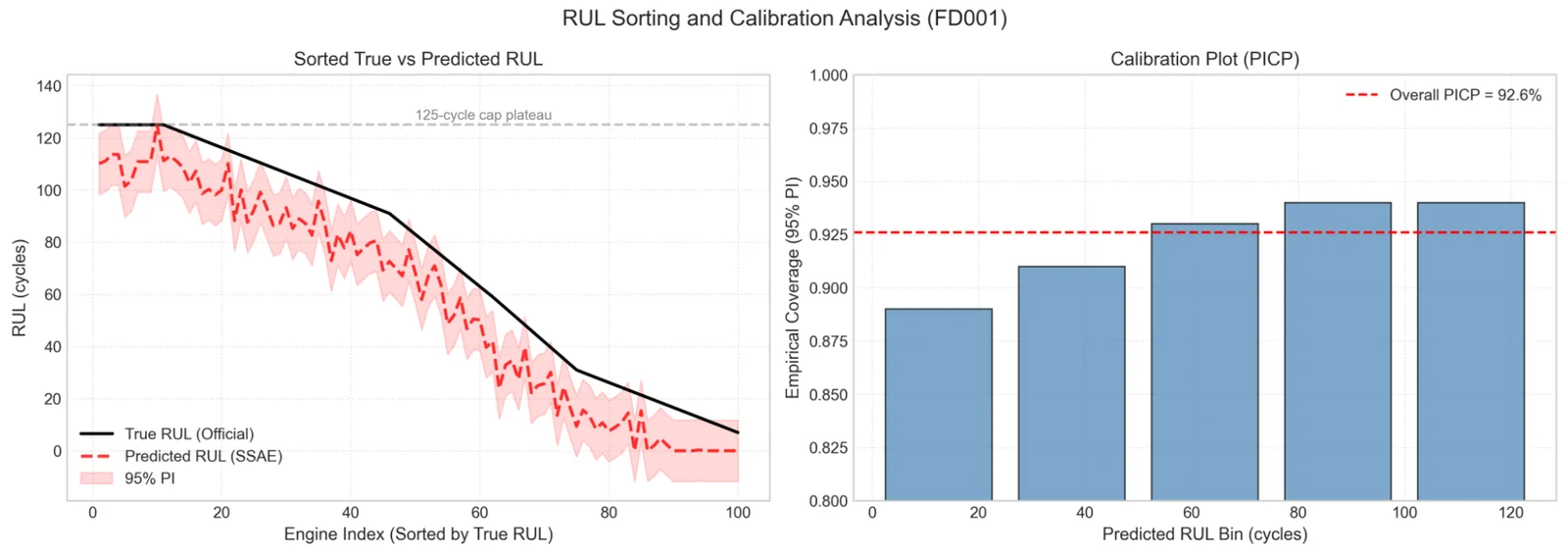
Sorted RUL predictions and interval coverage on FD001.

Ordering the engines by reference RUL in Figure 7 provides a population-level check of whether the model preserves the degradation structure across the test set. The predictive mean follows the descending profile from the 125-cycle capped region through the intermediate-RUL transition and into the near-failure regime, while the 95% prediction interval evolves with the same trajectory. Across successive predicted-RUL bins, the empirical coverage is approximately 0.89, 0.91, 0.93, 0.94, and 0.94, giving an aggregate PICP of 92.6%. The limited variation between bins indicates that the uncertainty estimate is not dominated by a single operating range. This calibration is particularly important for RAPM because CVaR is a tail functional: its value depends on both the location and dispersion of the predictive distribution. The observed coverage therefore supports direct propagation of the SSAE distribution into lower-tail risk estimation rather than the addition of a heuristic safety margin to a point prediction.

The selection of SSAE as the prognostic backbone is motivated by its structural properties. The sparse autoencoder enforces a compact latent representation that suppresses irrelevant sensor fluctuations, thereby improving robustness under varying operating conditions. The probabilistic output layer enables the joint estimation of predictive mean and uncertainty within a unified framework, avoiding inconsistencies associated with post hoc uncertainty modeling. In addition, the reconstruction objective acts as an implicit regularizer, enhancing generalization when the model is exposed to perturbed or noisy inputs.

**Figure 8:**
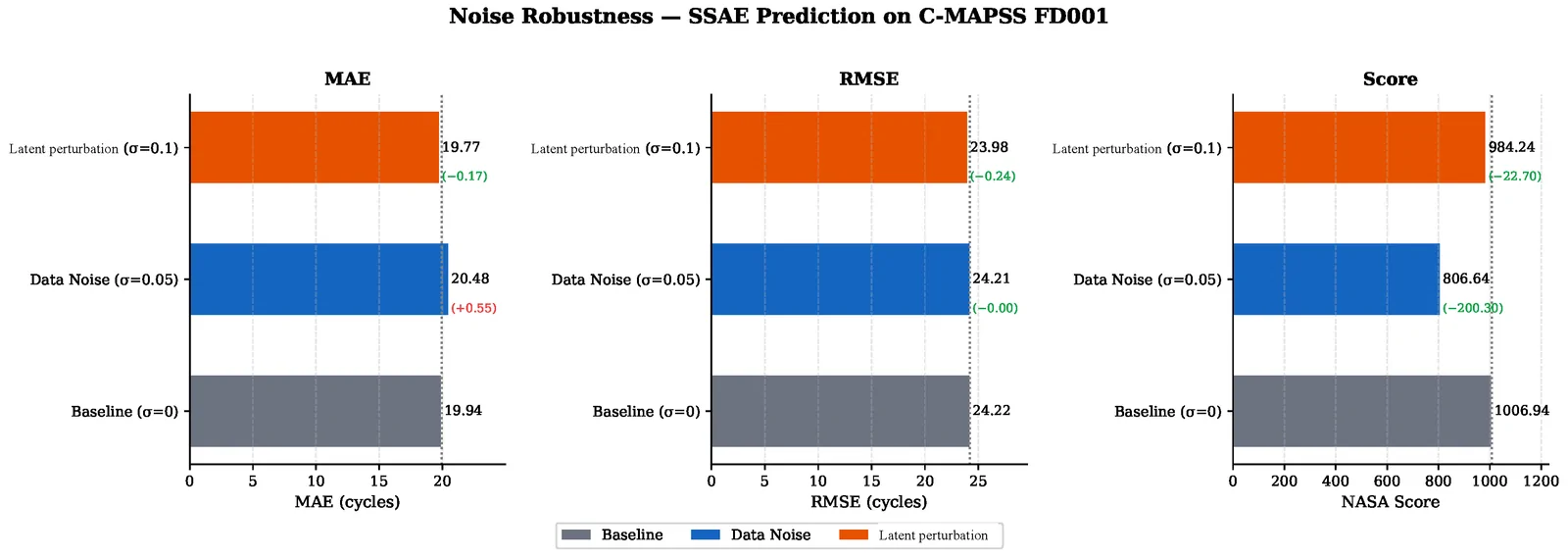
SSAE prediction under baseline conditions, sensor noise (σ=0.05), and latent perturbation (σ=0.10).

To examine sensitivity to controlled perturbations, Figure 8 evaluates the model under sensor noise and latent perturbation. Gaussian noise is injected into the normalized sensor inputs as a numerical stress test rather than as a substitute for measurements from a physical sensor rig, while latent perturbations are introduced within the SSAE encoder representation to assess sensitivity to model approximation or quantization effects. These perturbations are applied only within the benchmark evaluation protocol described here.

The robustness experiment holds the SSAE checkpoint fixed so that every perturbed result is paired with the same unperturbed prediction set. The corresponding baseline is an MAE of 19.94, an RMSE of 24.22, and a NASA Score of 1006.94. Its small difference from Table 4 arises because the table aggregates five independently trained models, whereas the paired design isolates perturbation sensitivity from training-seed variation.

The results demonstrate that the SSAE model maintains stable predictive performance under both types of perturbation. Under sensor noise with standard deviation σ=0.05, the MAE increases by approximately 0.5 cycles, corresponding to a relative change of about 2.6%, while the RMSE remains nearly unchanged. Under latent perturbations with σ=0.10, both MAE and RMSE exhibit negligible variation with occasional slight improvements attributable to stochastic smoothing effects. Although the NASA score decreases under data noise, this change is primarily driven by its asymmetric penalty for late predictions rather than a systematic degradation of predictive quality.

For RAPM, the relevant prognostic quantity is therefore a late-life-sensitive predictive distribution whose lower tail remains stable under controlled perturbations. This distribution, rather than the point estimate alone, is passed to the CVaR and semantic risk-abstraction modules before PPO optimization.

Table 1 relates representation capacity to the two validation objectives. Increasing *d* from 16 to 32 reduces reconstruction MSE from 0.041 to 0.032 and RUL MAE from 20.8 to 19.9 cycles. Increasing *d* to 64 further reduces reconstruction MSE to 0.029, while RUL MAE rises to 20.3 cycles. The 32-dimensional representation therefore achieves the lowest validation RUL error among the tested configurations.

The sample-size response is concentrated between N=20 and N=50. Increasing *N* from 20 to 50 changes the predictive mean by 0.1 cycles, increases total predictive standard deviation by 0.3 cycles, shifts pre-projection CVaR by 0.5 cycles, and raises PICP by 0.8 percentage points. The corresponding changes from N=50 to N=100 contract to 0.0, 0.1, 0.2 cycles, and 0.2 percentage points, respectively. Thus, N=50 captures the same distributional regime observed at N=100 while using half as many stochastic forward passes, and it was retained for all subsequent uncertainty and decision experiments.

### 4.2. Independent Maintenance Comparison and Backbone Generality

The decision-level comparison was designed to separate the performance of RAPM from methods that share its SSAE backbone. The external baselines in Table 5 employ independently trained prognostic models and distinct decision mechanisms, ranging from fixed-threshold intervention to probabilistic DRL and ensemble-based dynamic scheduling. All numerical values were produced by our independent implementations under the common out-of-fold protocol; the citations identify the originating methods and do not denote values copied from those publications. All methods were evaluated on the same held-out engines using common definitions of maintenance outcomes. Under the nominal horizon W=30, an intervention is classified as optimal when 1<RUL≤W, early when RUL>W, late when 0<RUL≤1, and failed when operation continues at RUL≤0.

RAPM achieves an optimal stopping rate of 96.0%, exceeding the strongest independent baseline, Ensemble-DTS, by 5.8 percentage points. The improvement is accompanied by a reduction in early maintenance from 8.0% to 4.0%, while no late interventions or failure events are recorded. RAPM also produces the lowest average RUL at stopping among the methods with zero failures, indicating a deeper utilization of the available service life within the prescribed maintenance window. Because the comparison includes fixed-threshold, ensemble-scheduling, and independently trained DRL approaches, the observed advantage is not tied to a shared prognostic backbone or a single decision paradigm.

Table 6 isolates the influence of the prognostic backbone while preserving the complete CVaR-LLM-PPO decision layer. For each configuration, the backbone was independently trained, the predictive mean and CVaR sequences were regenerated, and the PPO policy was retrained using the same fixed architecture, hyperparameters, curriculum, training budget, and random seeds. The optimal stopping rates range from 92.4% to 96.0% across the five probabilistic backbones, and none of the configurations produces a late intervention or failure. In particular, Transformer-MCD reaches 95.2%, compared with 96.0% for the probabilistic SSAE, despite their substantial difference in global point-prediction error. The 0.8-percentage-point difference is small relative to the run-to-run variation, and the two configurations therefore provide comparable decision-level performance. The controlled replacement confirms that the effectiveness of the CVaR-LLM-PPO layer is preserved when the prognostic backbone is changed.

The SSAE nevertheless records the highest mean optimal stopping rate and the lowest mean RUL at intervention among the evaluated configurations. This ordering is consistent with its probabilistic scores and late-life error profile: relative to Transformer-MCD, the SSAE reduces NLL from 0.41 to 0.39 and CRPS from 18.5 to 17.8 cycles, improves 95% PICP from 91.0% to 92.6%, and reduces the MAE within RUL≤30 from 13.8 to 13.1 cycles. These properties provide a more informative input distribution for CVaR near the intervention region even though they do not minimize global point error. The SSAE is therefore retained as the reference prognostic configuration on the basis of its distributional quality and decision-critical accuracy, while the consistently high performance of the alternative backbones establishes the transferability of the proposed decision architecture.

### 4.3. Validation of the Gaussian CVaR Approximation

**Figure 9:**
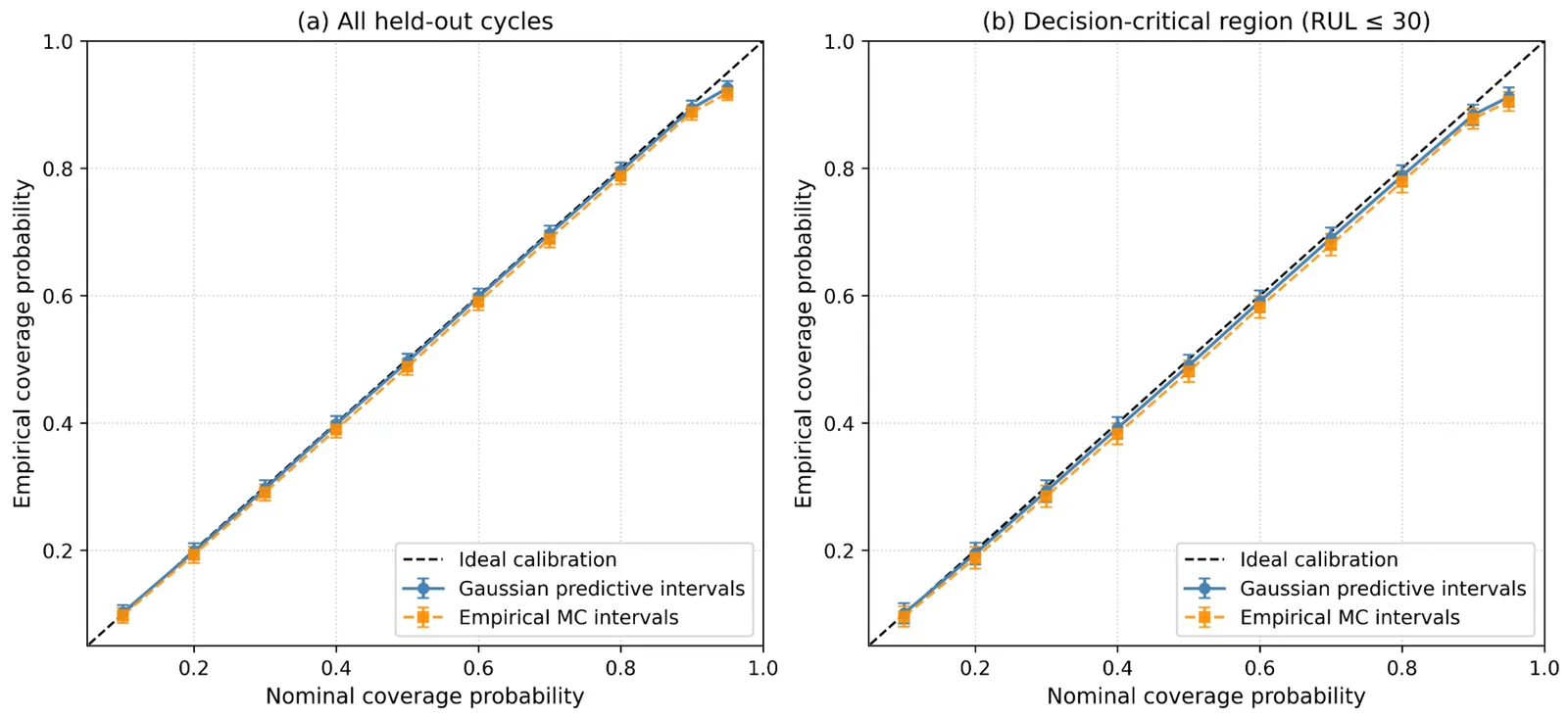
Reliability of Gaussian and empirical predictive RUL intervals.

Figure 9 establishes calibration across the predictive distribution rather than at a single coverage level. Over all held-out cycles, the Gaussian intervals attain a mean absolute calibration error of 0.016 and 92.6% empirical coverage at the nominal 95% level. The corresponding values in the decision-critical region (RUL≤30) are 0.022 and 91.2% with a maximum calibration error of 0.038. The empirical MC intervals follow the same calibration profile, demonstrating consistent uncertainty coverage under direct sample quantiles and moment-matched Gaussian intervals.

The cycle-wise distributional diagnostics in Table 7 resolve the shape of the MC predictive mixture. Median skewness increases from 0.42 over the complete evaluation set to 0.91 for RUL≤30, while median excess kurtosis increases from 0.85 to 1.72. At the 5% significance level, the Anderson–Darling test rejects normality for 28.0% of all cycles and 46.0% of decision-critical cycles. These diagnostics identify the strongest mixture asymmetry near end-of-life and motivate the direct validation of the lower-tail functional used by the maintenance policy.

**Figure 10:**
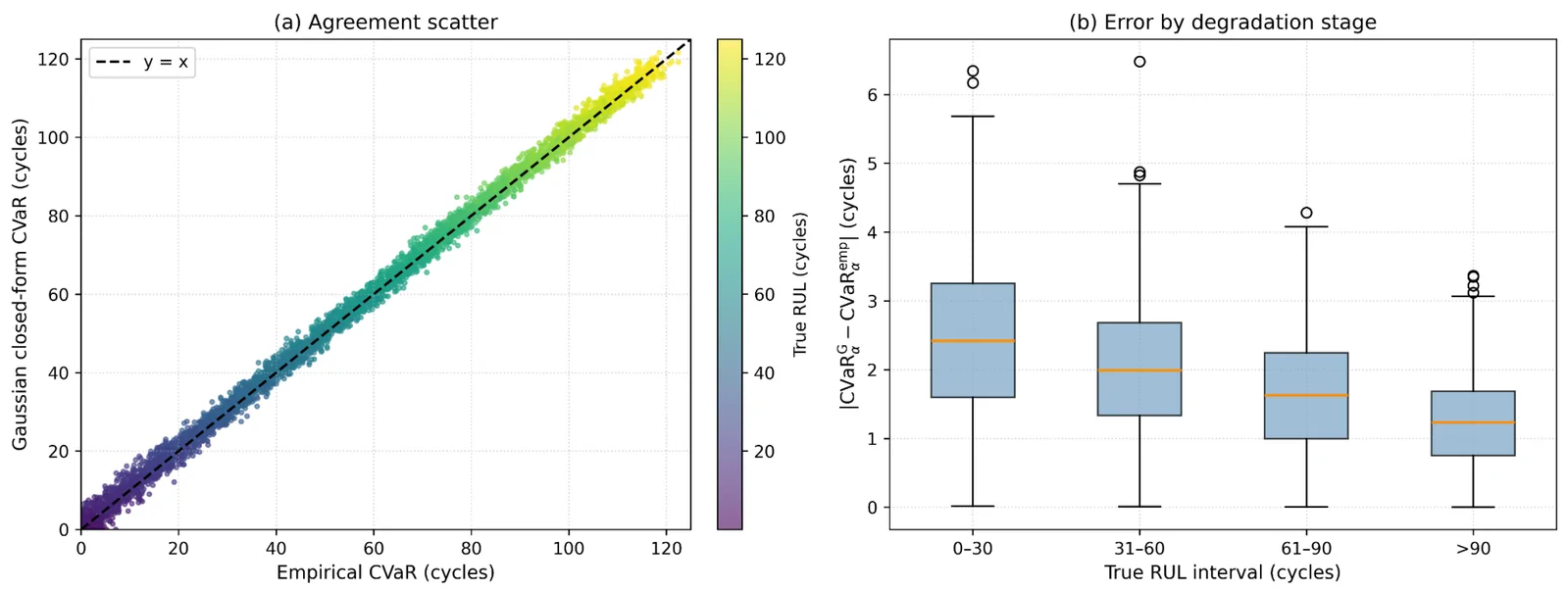
Agreement between Gaussian and empirical lower-tail CVaR estimates.

Figure 10 shows close cycle-level agreement between the two lower-tail estimators. The Gaussian estimate has a bias of +0.6 cycles and an MAE of 1.8 cycles over all held-out cycles; within RUL≤30, the corresponding values are +1.1 and 2.4 cycles. The 95th-percentile absolute discrepancy remains 4.1 cycles over the complete set and 4.8 cycles in the decision-critical region. The positive bias indicates a small upward displacement of the Gaussian lower-tail RUL, while the stage-wise error distribution remains concentrated across the degradation trajectory.

The frozen-policy substitution in Table 7 propagates the empirical estimator through the same physical constraint, cached semantic-risk mapping, and PPO policies used by the Gaussian estimator. Across matched held-out states, the estimator replacement changes 4.8% of semantic risk levels and 1.6% of actions. The optimal stopping rate changes from 96.0% to 95.8%, the mean RUL at intervention changes from 23.2 to 23.3 cycles, and both estimators retain zero late interventions and zero failures. The moment-matched Gaussian formulation therefore preserves the decision-relevant lower-tail structure of the MC predictive mixture and supports the maintenance conclusions obtained with Equation (5).

### 4.4. Bias-Controlled CVaR Ablation and Decision Robustness

Table 8 separates the systematic location error of the SSAE mean from the dispersion information carried by CVaR. Panel (a) reports the recalculated prognostic metrics on the official FD001 test split, and Panel (b) reports the matched out-of-fold maintenance experiment.

Validation-derived calibration shifts the official-test residual bias from −16.7 to +0.2 cycles. The corresponding MAE and RMSE decrease from 19.9 and 24.1 cycles to 13.9 and 17.4 cycles, respectively; the MAE within RUL≤30 decreases from 13.1 to 12.3 cycles, and 95% PICP increases from 92.6% to 93.8%. These changes are obtained with the original MC predictive variance, isolating the effect of the location correction.

Within the bias-corrected decision control, retaining CVaR raises the optimal stopping rate from 93.4% to 95.8%, reduces early intervention from 6.6% to 4.2%, and shifts the mean RUL at intervention from 23.8 to 23.3 cycles with zero late interventions and zero failures. The unprojected predictive mean and Gaussian CVaR undergo the same validation-derived translation, leaving $\Delta_{t}^{\mathrm{unc}}=2.063{\hat{\sigma }}_{t}$ invariant; the physical projection is applied subsequently to the corrected lower-tail estimate. Because both bias-corrected policies share the same calibrated location signal, the retained 2.4-percentage-point advantage identifies predictive dispersion as an independent contributor to stopping precision. Its proximity to the 3.0-percentage-point advantage before calibration further shows that the CVaR gain is not generated by the original negative mean bias.

Following this bias control, Table 9 isolates the contribution of CVaR within the matched Rule-PPO architecture under sensor and latent-feature perturbations. The Rule-PPO and No-CVaR variants use the same SSAE predictions, risk-mapping procedure, reward coefficients, and training schedule; the distinction is whether the lower-tail estimate and its associated dispersion signal are retained in the decision state. Random Policy is included as a sanity-check reference.

Table 10 quantifies the paired differences underlying Table 9. The baseline gain is 3.0 percentage points with a 95% confidence interval of [−0.6,+6.8] spanning zero. Under sensor noise, the gain reaches 9.0 percentage points with a strictly positive interval of [+4.8,+13.4]; under latent-feature perturbation, it is 4.0 percentage points with an interval of [+0.4,+7.8]. Both perturbation conditions therefore support a positive CVaR contribution to optimal stopping after accounting for engine and seed variation.

The terminal-RUL statistics distinguish the policies within their common zero-failure outcome. CVaR reduces $q_{<5}$ from 2.2% to 0.6% at baseline, from 5.8% to 1.4% under sensor noise, and from 2.8% to 0.8% under latent perturbation. Under sensor noise, the 4.4-percentage-point reduction corresponds to 75.9% fewer terminations below five cycles. This reduction accompanies a lower early-intervention rate (18.0% versus 27.0%) and a lower mean RUL at intervention (26.3 versus 27.8 cycles). CVaR thus concentrates more interventions within the maintenance window while combining deeper life utilization with fewer stops close to the failure boundary.

The CVaR-enabled Rule-PPO uses the state definition in Equation (9),

(14) $$s_{t}=\left[x_{f,t},{\hat{\mu }}_{t},{\mathrm{CVaR}}_{t},l_{t}\right].$$

The No-CVaR control replaces ${\mathrm{CVaR}}_{t}$ with ${\hat{\mu }}_{t}$ and sets $\Delta_{t}^{\mathrm{unc}}=0$ before risk mapping. Both the additional state coordinate and the rule-derived risk level therefore carry the same location-only prognostic information, isolating the contribution of predictive dispersion.

**Figure 11:**
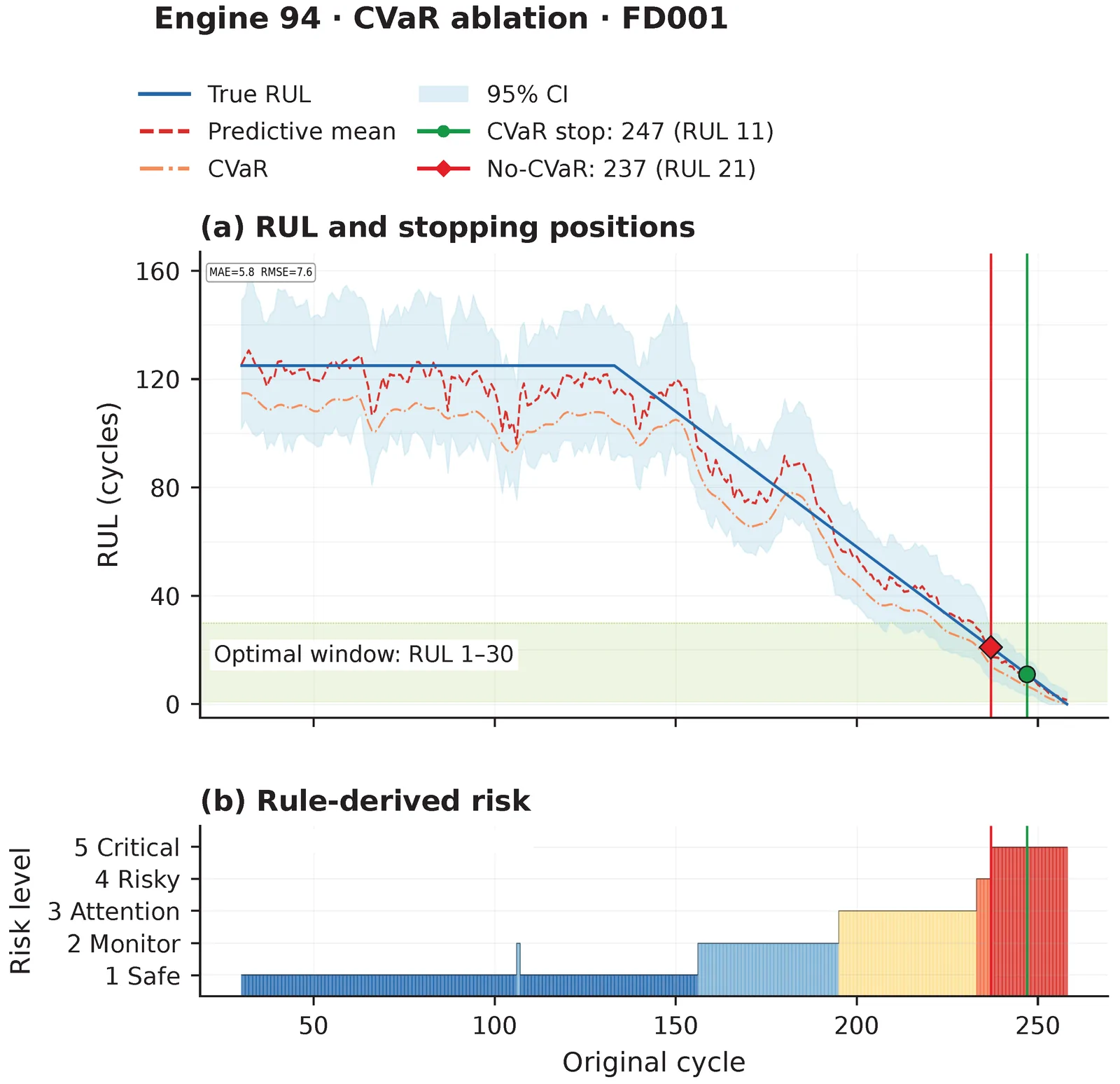
CVaR ablation on Engine 94. (**a**) Predictive mean, CVaR, true RUL, and the 95% interval; the shaded maintenance window spans RUL 1–30. The CVaR-enabled and No-CVaR policies stop at original cycles 247 and 237 with true RUL values of 11 and 21, respectively. Trajectory MAE and RMSE are 5.8 and 7.6 cycles. (**b**) Rule-derived ordinal risk with levels identified by bar height and labelled ticks.

To complement the aggregate statistics, Figure 11 provides a trajectory-level comparison of the stopping behavior induced by CVaR on a representative engine. The full Rule-PPO policy and its No-CVaR counterpart are evaluated on the same degradation trajectory, using the same SSAE predictive backbone, so that the only structural difference lies in whether the lower-tail risk estimate is retained in the observation and risk evaluation pipeline. In the implementation of the ablated variant, the conservative estimate is forcibly replaced by the predictive mean, i.e., ${\mathrm{CVaR}}_{t}={\hat{\mu }}_{t}$, and the dispersion input is set to $\Delta_{t}^{\mathrm{unc}}=0$. In contrast, the full policy preserves the joint state representation that includes both ${\hat{\mu }}_{t}$ and ${\mathrm{CVaR}}_{t}$ together with the derived risk level.

Figure 11 shows that the No-CVaR policy stops at cycle 237 with 21 cycles of true RUL, whereas the CVaR-enabled policy stops at cycle 247 with 11 cycles of true RUL. The latter retains ten additional operating cycles while intervening within the same maintenance window. The joint mean–CVaR state gives PPO access to both predictive location and lower-tail dispersion, allowing the learned policy to condition intervention timing on their combined evolution. The paired trajectory therefore illustrates the greater residual-life utilization associated with the CVaR-enabled decision state.

The trajectory-level evidence in Figure 11 is fully consistent with the aggregate results in Table 9. At the population level, the inclusion of CVaR increases the optimal stopping rate while preserving zero failure events; at the individual level, it shifts the stopping point deeper into the allowable maintenance window without sacrificing safety. This combination is particularly important for predictive maintenance, where the practical objective is not merely to avoid failure but rather to exploit as much residual life as possible before intervention. The Engine 94 example therefore provides a mechanistic interpretation of the quantitative gains reported in Table 9: the benefit of CVaR arises from improved timing precision rather than from a trivial increase in caution.

**Figure 12:**
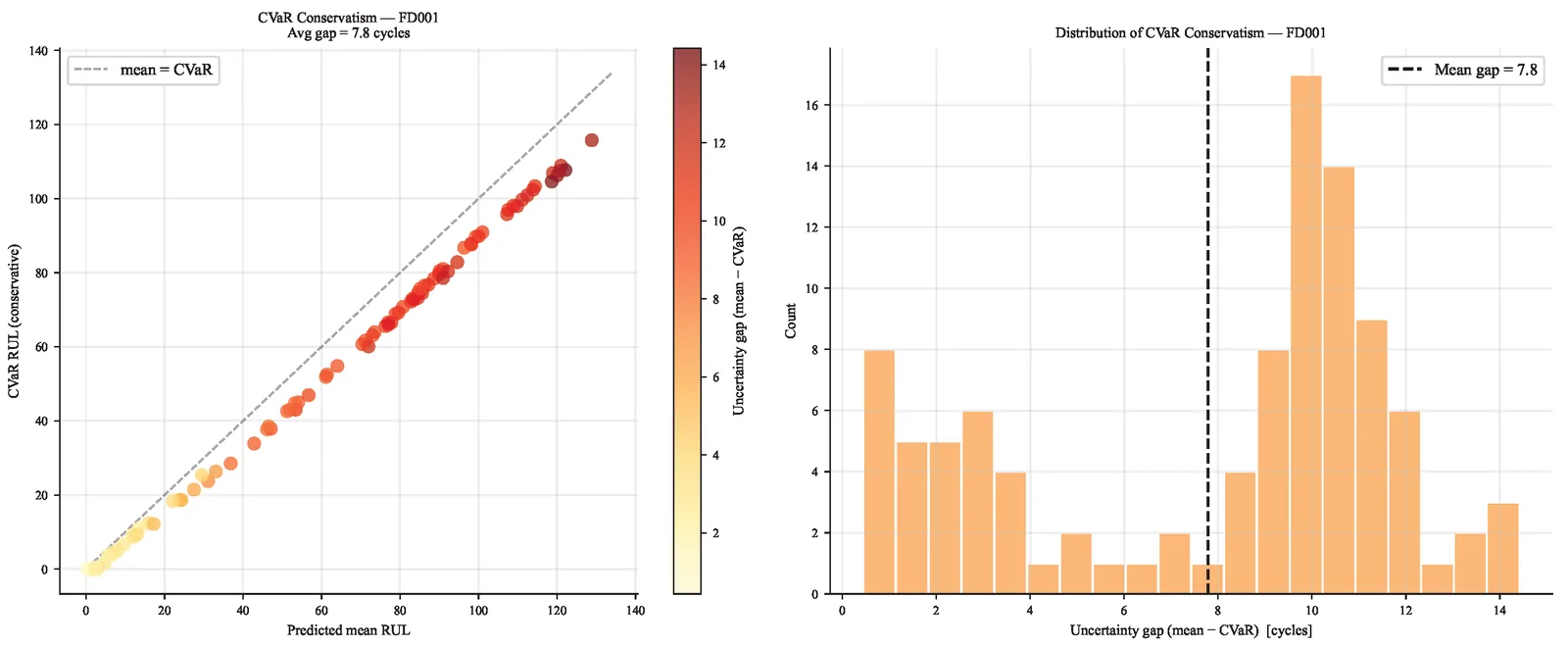
Projected CVaR versus predictive mean RUL and the distribution of their difference on FD001.

Figure 12 characterizes the conservative RUL signal supplied to the policy. The projected CVaR values lie at or below the corresponding predictive means, and the mean projected gap is 7.8 cycles. This figure reports ${\hat{\mu }}_{t}-{\mathrm{CVaR}}_{t}$, whereas semantic uncertainty is represented by the unprojected gap $\Delta_{t}^{\mathrm{unc}}={\hat{\mu }}_{t}-{\mathrm{CVaR}}_{\alpha ,t}^{G}$ in Equation (7). When the Gaussian lower-tail estimate reaches zero, physical projection compresses the plotted gap while the unprojected quantity retains the full dispersion signal. The trajectory-level effect on intervention timing is shown separately in Figure 11.

The robustness of this mechanism is further examined under noisy conditions. Two perturbation scenarios are considered: sensor noise applied directly to the inputs and latent perturbation applied to the SSAE encoder representation. In both cases, the noise is introduced at the level of the observation pipeline, thereby influencing both prediction and decision processes.

The results demonstrate that the CVaR-based policy maintains stable decision quality under both perturbation types. While the failure rate remains zero across all configurations, the optimal stopping rate degrades more slowly compared to the No-CVaR baseline. In particular, under data noise, the absence of CVaR leads to a pronounced increase in early stopping events, reflecting the policy’s inability to distinguish between noise-induced fluctuations and genuine degradation signals. By contrast, the CVaR-enabled policy exhibits greater resilience, as the lower-tail estimate naturally filters out the optimistic bias introduced by noise.

A similar trend is observed under latent perturbation. When noise is injected into the latent space, the predictive variance increases, but the CVaR mechanism translates this increase into a more conservative decision signal. Consequently, the policy adapts its behavior in a manner that preserves decision reliability. This adaptive response is absent in the No-CVaR variant, where the lack of uncertainty awareness results in higher variability in stopping behavior.

**Figure 13:**
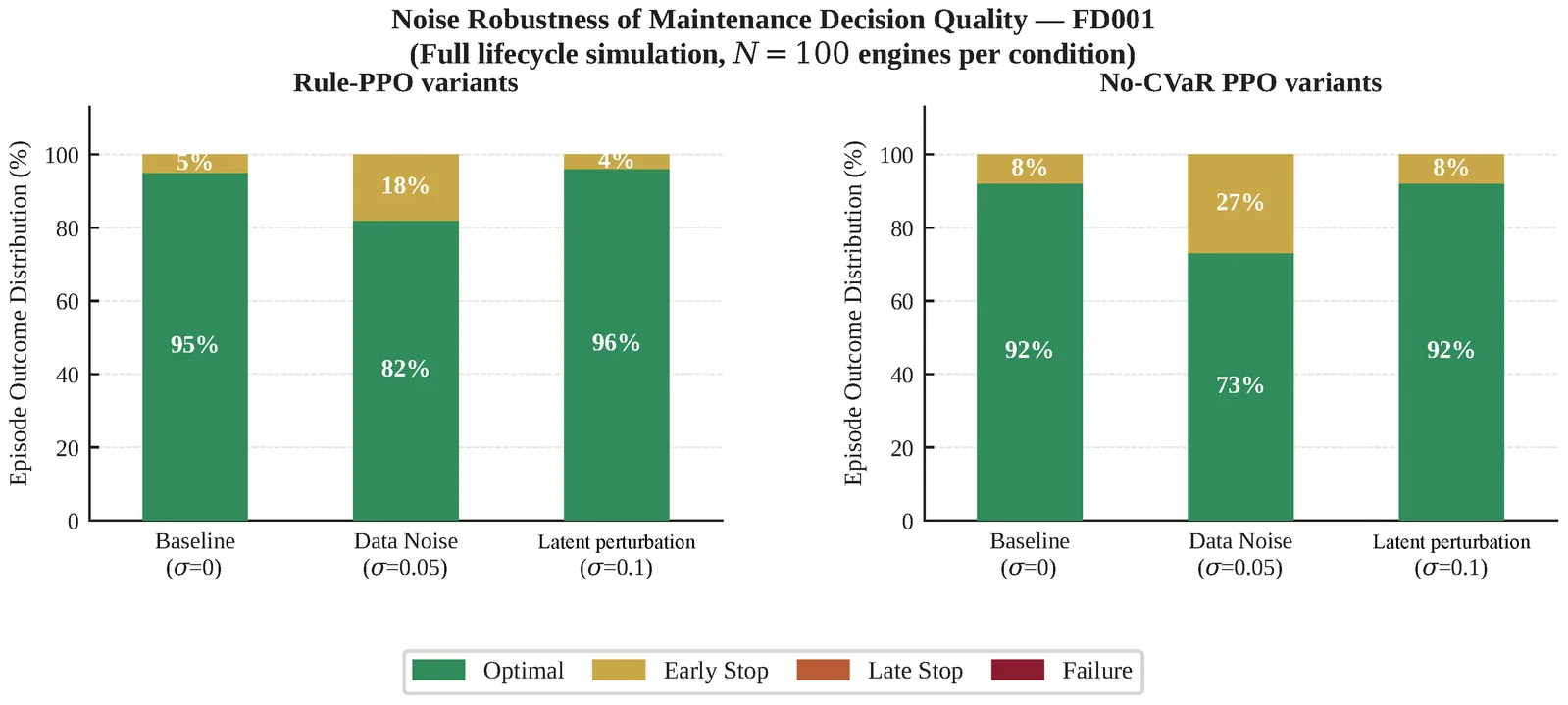
Decision outcome distributions under baseline conditions, sensor noise (σ=0.05), and latent perturbation (σ=0.10).

Figure 13 presents the distribution of maintenance outcomes under baseline, sensor-noise, and latent-perturbation conditions for both the Rule-PPO and No-CVaR PPO variants. A clear distinction emerges in the proportion of optimal and early stopping decisions. Under baseline conditions, the CVaR-enabled policy achieves an optimal stopping rate of 95% compared with 92% for the No-CVaR variant. When data-level noise is introduced, the optimal stopping rate drops to 82% for Rule-PPO and to 73% for No-CVaR PPO, while the proportion of early interventions increases from 5% to 18% and from 8% to 27%, respectively. This result indicates that the absence of CVaR makes the policy substantially more vulnerable to noisy observations, causing it to interpret noise-induced fluctuations as signs of imminent degradation. Under latent perturbation, both policies recover close to their baseline performance with Rule-PPO reaching 96% optimal stopping and No-CVaR PPO remaining at 92%. This suggests that the decision framework is less sensitive to latent perturbations than to the direct corruption of the sensor inputs.

**Figure 14:**
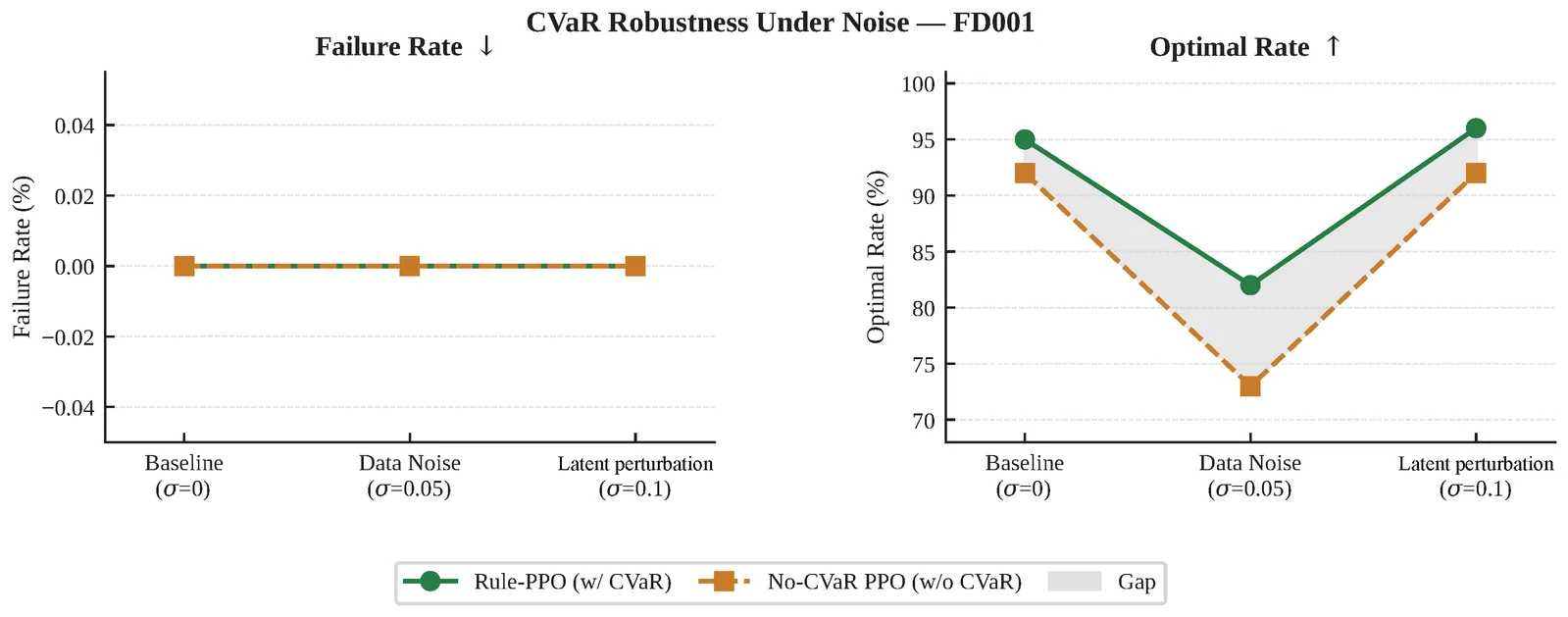
Robustness of CVaR-based maintenance decisions under sensor noise (σ=0.05) and latent perturbation (σ=0.10).

Figure 14 summarizes the observed failure rates and optimal stopping rates. The paired intervals and terminal-RUL frequencies in Table 10 resolve the distinction between the two policies: sensor noise produces the largest observed difference in optimal stopping, which are accompanied by fewer terminations within five cycles of failure. Together with the reduced early-intervention rate in Figure 13, these results link the CVaR signal to more precise maintenance timing across both ends of the intervention window.

### 4.5. Stopping-Window Sensitivity and Curriculum Ablation

Table 11 separates metric reclassification from policy adaptation. Freezing the nominal W=30 policy leaves every action and the mean intervention RUL unchanged at 23.2 cycles. Tightening the evaluation window to W=20 reclassifies stops in the 21–30-cycle interval as early and reduces the optimal rate to 30.0%, whereas widening it to W=40 raises the optimal rate to 99.0%. Neither reclassification introduces a late intervention or failure. Under matched retraining, the policy preserves optimal stopping rates of 94.0%, 96.0%, and 95.2% for W=20, 30, and 40, respectively, with zero late interventions and failures throughout. The W=20 policy moves the mean intervention RUL to 16.5 cycles, yielding later stopping and deeper residual-life utilization; the W=40 policy moves it to 30.8 cycles, yielding earlier stopping and a longer maintenance lead time. The nominal W=30 setting therefore occupies an intermediate operating point between these two planning horizons.

**Figure 15:**
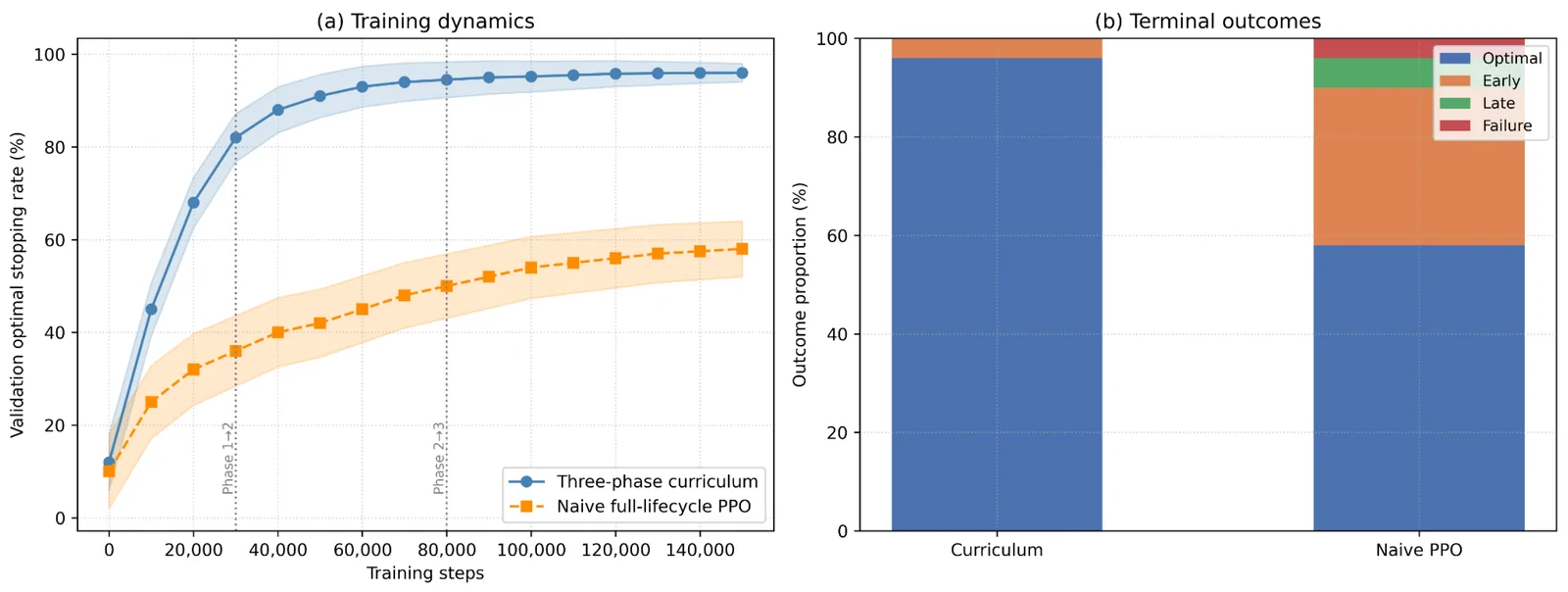
Curriculum ablation: training dynamics and terminal outcomes.

Table 12 and Figure 15 isolate the training effect of progressive horizon expansion. The curves report the mean validation optimal-stopping rate over five seeds with shaded bands denoting one standard deviation. The three-phase policy reaches 96.0±2.1%, whereas naive full-lifecycle PPO reaches 58.0±5.2% under the same 150k-transition budget. Its dominant error is premature intervention (32.0±4.8%), which is accompanied by 6.0±2.1% late interventions and 4.0±1.8% failures. The terminal outcome composition in Figure 15b shows that exposing the untrained policy directly to complete trajectories produces a diffuse stopping boundary, while progressive horizon expansion concentrates decisions inside the target window and removes the late and failure modes. Curriculum learning thus provides the optimization pathway required to translate the risk-aware state into stable long-horizon maintenance decisions.

### 4.6. Effect of LLM-Based Risk Interpretation on Stopping Behavior

**Figure 16:**
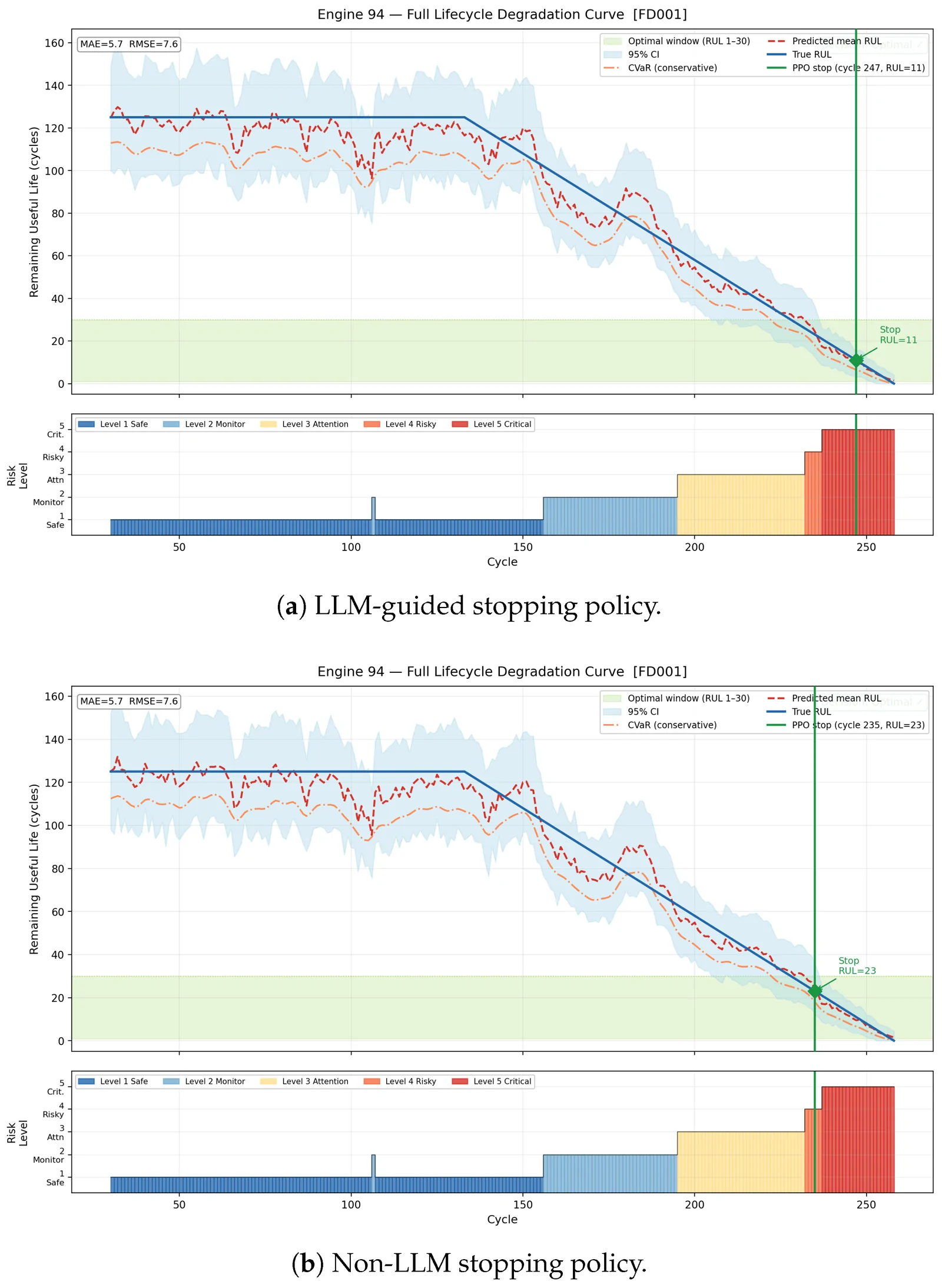
Single-engine comparison of stopping behavior with and without the LLM risk layer.

To further examine the role of the LLM module at the decision level, Figure 16 compares the stopping behavior of the full LLM-PPO policy and its non-LLM counterpart on the same engine trajectory. In both cases, the underlying prognostic backbone remains unchanged: the same SSAE model provides the predicted mean RUL, the same uncertainty band is propagated through the CVaR estimate, and the same degradation process is observed. The difference therefore lies not in the predictive signal itself but rather in the way that signal is interpreted and converted into decision guidance. Holding ${\hat{\mu }}_{t}$, ${\mathrm{CVaR}}_{t}$, and the underlying SSAE predictions fixed isolates the mapping $c_{t}\mapsto l_{t}$; the resulting change in stopping time therefore arises from semantic risk abstraction rather than from a different numerical RUL estimate.

The comparison reveals a clear shift in stopping behavior. With the LLM module enabled, the policy delays maintenance until cycle 247, where the true remaining useful life is 11 cycles. Without the LLM module, the policy stops earlier at cycle 235, corresponding to a true remaining useful life of 23 cycles. Both decisions remain inside the admissible maintenance window, but the LLM-guided policy exploits a larger fraction of the residual life before intervention. This result suggests that the LLM does not merely replicate the numerical risk signal; rather, it reshapes the timing of the final decision by providing an additional semantic interpretation of degradation severity.

This behavioral difference is consistent with the design of the LLM risk layer. In the implementation, the language model does not output a maintenance action directly. Instead, it converts the current prognostic state, including the predicted RUL and conservative lower-tail estimate, into a discrete risk level that is then consumed by the reinforcement learning policy as part of the state representation and reward-shaping mechanism. The comparison scripts further show that the same engine trajectory can be re-evaluated under different risk interpretation modules while holding the predictive model fixed, making it possible to isolate the influence of semantic risk abstraction on stopping time.

From the perspective of decision dynamics, the non-LLM policy is more tightly coupled to the raw numerical signal and therefore tends to respond earlier once the predicted trajectory enters the optimal maintenance window. The LLM-guided policy, by contrast, appears to tolerate a longer interval of late-stage degradation before issuing the stop decision. This indicates that the semantic risk layer provides a different notion of urgency than a purely rule-based interpretation. Rather than reacting directly to threshold crossings, the LLM-mediated policy evaluates the degradation state in a more context-dependent manner, which in this example translates into a later and more precise intervention.

It is important to note that this case study is not intended to claim universal superiority from a single trajectory. Its value lies in illustrating the mechanism by which the LLM component affects policy behavior. Under an identical predictive trajectory, the presence of the LLM shifts the stopping point deeper into the allowable maintenance window, thereby increasing residual-life utilization without violating the safety constraint. In this sense, the contribution of the LLM module is not to alter the prognostic estimate itself but to modify how that estimate is operationally interpreted in the final control loop.

Taken together, Figure 16 suggests that the LLM layer acts as more than an auxiliary explanation tool. Even when the numerical prediction and CVaR profile are unchanged, semantic risk abstraction can still influence the timing of the stop decision. This provides further evidence that the proposed RAPM framework benefits not only from uncertainty-aware prediction but also from an interpretable semantic interface between prognostics and control.

### 4.7. Risk-Level Evolution and Validation of the LLM-Based Risk Design

To further assess whether the proposed risk design is effective, this subsection examines the evolution of the discrete risk signal and its influence on the final stopping decision. The emphasis here is not on prediction accuracy itself but on whether the risk abstraction layer can meaningfully reshape the control policy. In this respect, the most informative evidence is provided by the comparison between the rule-based risk agent and the LLM-based risk agent on the same engine trajectory.

**Figure 17:**
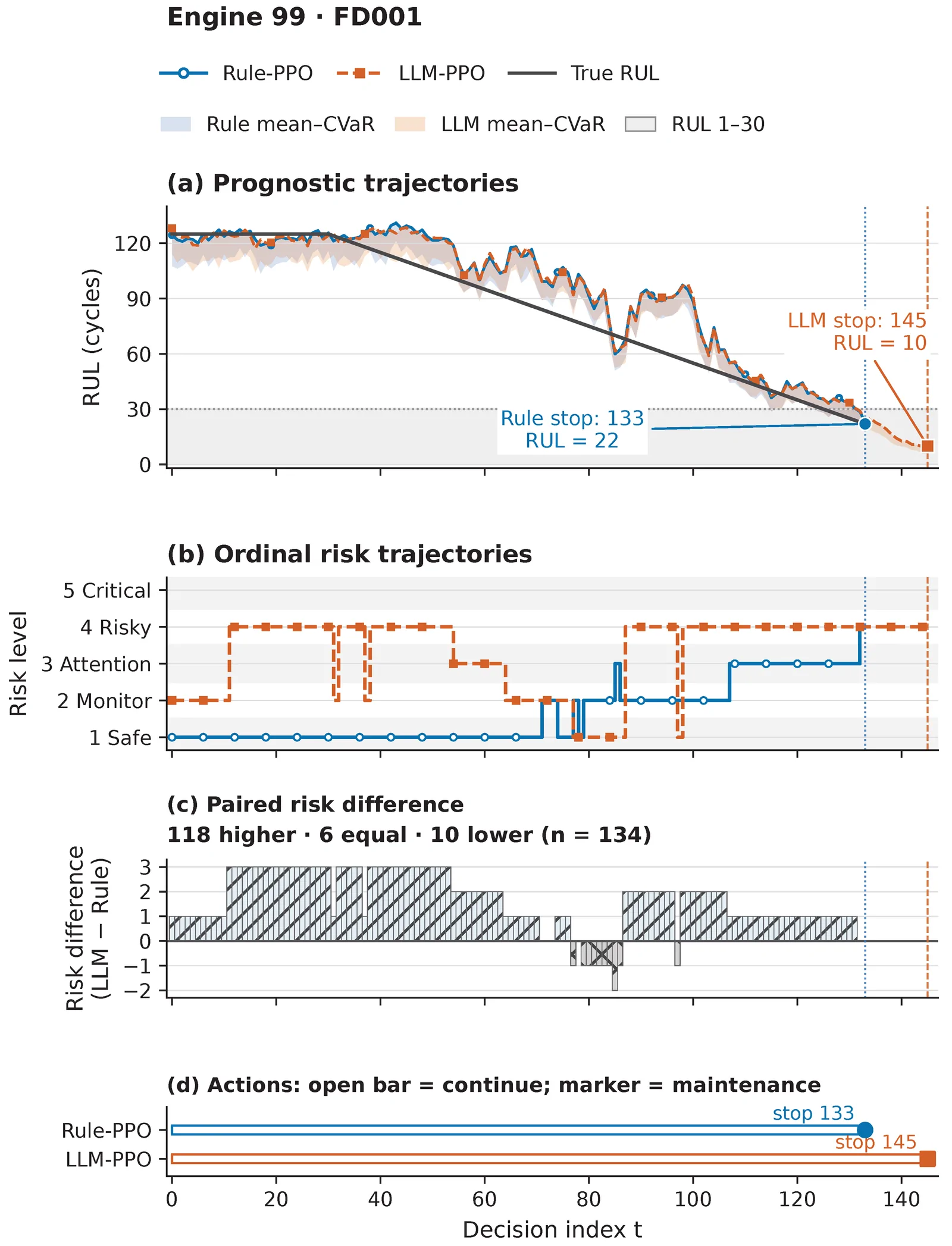
Risk-level comparison on Engine 99. (**a**) Predictive means, mean–CVaR bands, true RUL, and maintenance stops. (**b**) Ordinal risk: Rule-PPO uses a solid line with open circles, and LLM-PPO a dashed line with squares. (**c**) Paired risk differences over decision indices 0–133, inclusive; positive and negative differences use distinct hatching. (**d**) Continue/maintenance actions. Stop positions use the decision-index coordinate; risk levels are identified numerically.

Table 13 compares the risk levels on the 134 shared decision steps of Engine 99, from index 0 through the Rule-PPO stopping index 133, inclusive. The LLM assigns a higher level on 118 steps, the same level on 6 steps, and a lower level on 10 steps. The respective proportions are 88.1%, 4.5%, and 7.5%, which are each calculated with the common denominator 134. These paired counts agree with Figure 17 and identify a predominantly higher ordinal urgency under the LLM mapping.

This behavior is consistent with the architecture of the proposed framework. The rule-based risk agent maps ${\hat{\mu }}_{t}$ and ${\mathrm{CVaR}}_{t}$ into discrete levels through a small set of fixed thresholds, including explicit dependence on the dispersion gap $\Delta_{t}^{\mathrm{unc}}$. In contrast, the LLM-based agent is designed as a semantic abstraction layer rather than a thresholding module: it receives the same prognostic quantities but produces a contextualized risk judgment, which is then embedded into the observation and reward-shaping pipeline of PPO. The purpose of this design is precisely to test whether a semantically informed notion of risk can provide more useful guidance than a purely hand-crafted rule set.

Figure 17 illustrates this mechanism on Engine 99. The upper panel shows that the rule-based and LLM-based policies operate on nearly identical degradation trajectories and comparable uncertainty bands, so the difference in stopping behavior cannot be attributed to a different prognostic backbone. Instead, the divergence appears in the risk-level trajectory and in the accumulated risk difference. Across most of the lifecycle, the LLM assigns a higher risk level than the rule-based agent with the figure reporting 118 higher, 6 equal, and 10 lower assignments over the common timesteps. This confirms that the LLM consistently shifts the risk function upward.

Importantly, this upward shift does not translate into earlier stopping. The LLM-guided policy stops later, at plotted decision index 145 with a true RUL of 10 cycles, whereas the rule-based policy stops at index 133 with a true RUL of 22 cycles. Both decisions remain within the admissible maintenance window, but the LLM-based policy utilizes a larger fraction of the residual life. This result is important because it shows that the proposed risk design is not simply “more conservative” in the naive sense of stopping earlier. Instead, the LLM produces a denser and more persistent representation of risk throughout the degradation trajectory, allowing the PPO policy to internalize risk gradually rather than react only when a fixed threshold is crossed.

This interpretation is consistent with the reward design of the environment. The policy is not driven directly by the risk level alone; rather, the risk signal modulates the step reward through additional penalties at higher risk levels, while the terminal reward still favors stopping inside the optimal window. Consequently, a chronically higher LLM-based risk signal does not force immediate intervention. Instead, it reshapes the optimization landscape seen by PPO and changes how urgency is accumulated over time. The later stopping behavior observed in Figure 17 should therefore be understood as evidence that the LLM-based risk function provides a more informative temporal structure for decision making rather than a simple bias toward aggressive maintenance.

**Figure 18:**
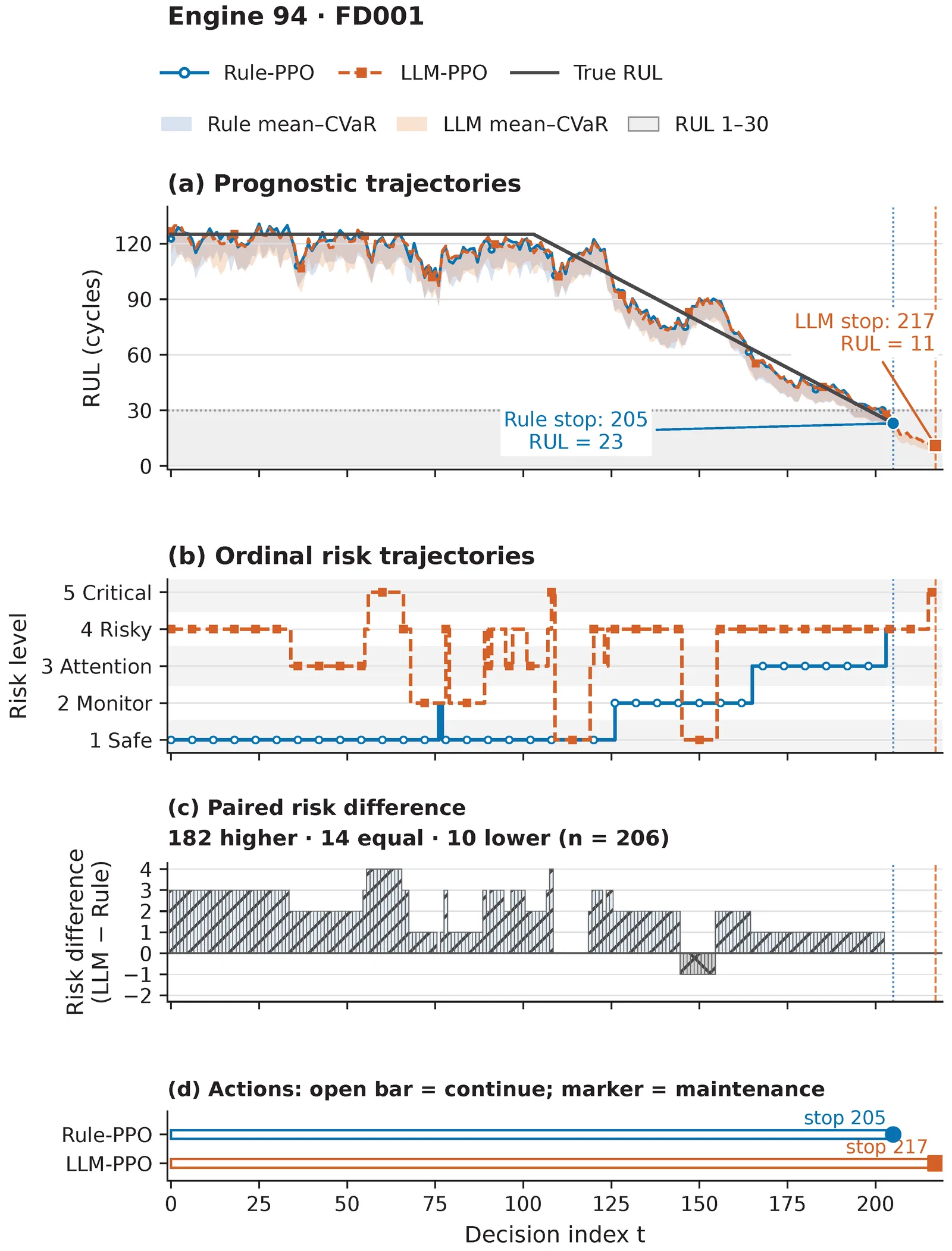
Risk-level comparison on Engine 94 using the line styles, markers, and hatching defined in Figure 17. Panels show (**a**) prognostic trajectories and maintenance stops, (**b**) ordinal risk, (**c**) paired risk differences, and (**d**) actions. Paired counts use decision indices 0–205, inclusive. True RUL provides the common life scale for comparison with Figure 16.

A second trajectory as shown in the Figure 18, Engine 94, exhibits the same qualitative trend. At the plotted decision indices 205 and 217, the rule-based and LLM-guided policies intervene with true RUL values of 23 and 11 cycles, respectively. These are the same intervention RUL values illustrated in Figure 16, whose horizontal axis uses the original cycle coordinate. Figure 18 reports 182 higher, 14 equal, and 10 lower LLM assignments across the 206 shared decision steps. Although these single-engine comparisons are not intended as statistical proof on their own, they provide consistent mechanistic evidence that the proposed risk design is functioning as intended across different degradation trajectories.

Taken together, Table 13, Figure 17 and Figure 18 support two conclusions. First, the LLM layer is effective in altering the shape of the risk function, making the risk representation more persistent and semantically sensitive than a fixed rule-based mapping. Second, this altered risk function is operationally useful: once coupled with PPO, it leads to later yet still safe interventions, thereby improving residual-life utilization. These results validate the central design claim of this work, namely that risk in predictive maintenance should not be represented solely as a static thresholding problem but rather as a structured semantic signal that evolves along the degradation trajectory.

### 4.8. Computational Efficiency

The computational efficiency of RAPM was evaluated on a workstation equipped with an Intel Core i9-13900K CPU, an NVIDIA RTX 4090 GPU with 24 GB memory, and 128 GB system memory (Santa Clara, CA, USA). Qwen2.5-3B [14] was deployed locally through Ollama using Q4_K_M quantization. All measurements used a batch size of one and the trained checkpoints employed in the maintenance experiments. After 50 warm-up iterations, component latency was recorded over 5000 decision cycles sampled from the 100 FD001 test trajectories. CUDA synchronization was applied before and after timing the PyTorch 2.14 modules, while LLM latency was measured end-to-end through the local Ollama interface. Median and 95th-percentile latency are reported to account for the right-skewed generation time of the language model.

The trainable part of RAPM remains compact: the probabilistic SSAE and the PPO actor–critic contain 0.30 M and 2.5 K trainable parameters, respectively. Qwen2.5-3B is loaded in quantized form and remains frozen throughout the experiments; it therefore contributes inference cost but introduces no additional optimization parameters. On the specified hardware, a single SSAE training run required 28 min. After the fixed-trajectory SSAE, CVaR, and validated semantic-risk sequences had been generated and cached once, the 150,000-step PPO optimization stage required 2.6 h. This duration refers to actor–critic optimization with cached upstream states and therefore is not obtained by multiplying the online per-cycle latency by the number of PPO steps. Peak memory usage during end-to-end execution was 4.2 GB on the GPU and 9.6 GB in system memory.

Let $N_{\mathrm{cycle}}$ denote the total number of decision cycles and $N_{\mathrm{LLM}}$ the number of fresh language-model queries. The effective query rate and avoided-query ratio are defined as

$$p_{q}=\frac{N_{\mathrm{LLM}}}{N_{\mathrm{cycle}}},\;\eta_{\mathrm{avoid}}=1-p_{q}.$$

Under the zero-based convention ${\mathrm{RUL}}_{t}=T-1-t$, the mean FD001 trajectory length of 206.31 cycles and the observed mean stopping RUL of 23.2 cycles correspond to 183.1 evaluated decision cycles per episode. Call-count profiling was therefore performed over 500 held-out engine–seed episodes (100 engines and five policy seeds) with cycle-wise invocation as the reference configuration.

Table 14 shows that cycle-wise invocation would require 91,550 fresh LLM calls, whereas the complete scheduler requires 2800, or $p_{q}=3.1\%$ of the reference call volume. Throttling accounts for the dominant reduction, and semantic caching further suppresses repeated evaluations of previously encountered RUL–CVaR states, yielding a 96.9% reduction and 5.6±0.8 fresh calls per episode. Using the measured component medians, the representative amortized latency is

$$\begin{array}{cc} {\tilde{\tau }}_{\mathrm{RAPM}}= & \tau_{\mathrm{SSAE}}^{\left(50\right)}+\tau_{\mathrm{CVaR}}+\tau_{\mathrm{PPO}}+p_{q}\tau_{\mathrm{LLM}}+\left(1-p_{q}\right)\tau_{\mathrm{reuse}}\\ = & 218.5+0.12+0.21+0.031\times 842.6+0.969\times 0.08\\ = & 245.0\;\mathrm{ms}. \end{array}$$

The single-pass SSAE latency in Table 15 is reported only as a profiling reference and is not added to the complete inference path, because both the predictive mean and uncertainty are obtained from the same 50 stochastic forward passes. These passes dominate the numerical path, whereas CVaR construction and PPO action selection together account for approximately 0.15% of its latency. Although an individual LLM query is more expensive than the numerical modules, its amortized contribution is only 26.1 ms per decision cycle because fresh queries occur sparsely. For comparison, cycle-wise LLM invocation would produce a representative latency of 1061.4 ms per cycle. Query throttling and semantic caching therefore reduce this value by 76.9%, to approximately 245.0 ms, while retaining the semantic risk layer within the maintenance loop.

## 5. Discussion

### 5.1. Design Trade-Offs and Limitations

The controlled backbone replacement clarifies the distinction between global point accuracy and decision utility. Using the five-seed statistics in Table 4, Transformer-MCD reduces the global MAE from 19.9±0.5 to 15.8±0.4 cycles relative to the probabilistic SSAE, yet the corresponding optimal stopping rates remain comparable at 95.2% and 96.0%, respectively. The different ranking arises because the maintenance policy does not act on the predictive mean alone. It also receives the dispersion-dependent CVaR estimate and the resulting semantic risk signal. Within this decision pathway, the SSAE provides the lowest NLL and CRPS, the interval coverage closest to the nominal 95% level, and the lowest MAE in the RUL≤30 region. These characteristics support its selection as the reference probabilistic backbone more directly than global MAE. At the same time, the 92.4–95.2% optimal stopping rates obtained with four alternative backbones demonstrate that the principal decision benefit of RAPM is transferable across prognostic architectures rather than being produced by one specific feature extractor.

The reliability profiles and empirical lower-tail comparison establish the calibration and decision relevance of the uncertainty signal supplied to RAPM. The moment-matched Gaussian CVaR differs from its empirical counterpart by 1.8 cycles in MAE over all held-out cycles and by 2.4 cycles in the decision-critical region. Under frozen-policy estimator substitution, this discrepancy changes only 1.6% of maintenance actions while preserving the optimal stopping rate, zero late interventions, and zero failures. The Gaussian formulation therefore provides a compact and decision-consistent representation of the predictive lower tail.

The MC sample-size analysis reaches the same conclusion from a computational perspective: increasing *N* from 50 to 100 changes pre-projection CVaR by only 0.2 cycles and PICP by 0.2 percentage points while doubling the number of stochastic forward passes. This stability supports N=50 as the operating point for the complete RAPM pipeline.

The additive-calibration control further separates this lower-tail contribution from the SSAE’s systematic location error. Removing the −16.7-cycle mean bias improves point accuracy while leaving the pre-projection dispersion gap invariant. Under this corrected input, CVaR Rule-PPO retains a 95.8% optimal stopping rate compared with 93.4% for the mean-only policy and reduces early intervention from 6.6% to 4.2%. The decision gain therefore follows the predictive dispersion encoded by CVaR rather than the negative offset of the original mean trajectory.

Paired inference supports the contribution of CVaR under perturbed observations. The sensor-noise gain of 9.0 percentage points has a 95% confidence interval of [+4.8,+13.4], and the latent-perturbation gain of 4.0 points has an interval of [+0.4,+7.8]. The accompanying reduction in $q_{<5}$ connects improved stopping precision to a lower frequency of interventions close to the failure boundary. Under sensor noise, this frequency decreases by 75.9% while average intervention RUL also decreases, showing that improved life utilization and a stronger lower-tail safety margin are achieved together.

The stopping-window and curriculum controls distinguish robustness to the planning horizon from optimization of the long-horizon policy. Matched retraining preserves 94.0–96.0% optimal stopping across W∈{20,30,40} while shifting the intervention point in the direction imposed by each horizon and maintaining zero late interventions and failures. By contrast, removing progressive horizon expansion reduces the optimal rate from 96.0% to 58.0% and introduces both late interventions and failures under the same transition budget. The resulting performance pattern attributes the stable decision boundary to the curriculum schedule rather than to a benchmark-specific choice of *W*.

The Qwen2.5-3B model occasionally produces inconsistent risk levels for similar inputs due to the stochastic nature of token sampling (temperature =0.1 mitigates but does not eliminate this). The five-cycle-bin semantic caching and the discrete {1,…,5} output space limit the practical impact of inconsistency, but a more deterministic risk mapping—such as a fine-tuned classifier trained on LLM-labeled data—would provide stronger guarantees for safety-critical deployment.

### 5.2. Benchmark Scope and Practical Deployment Considerations

The local deployment of Qwen2.5-3B via Ollama demonstrates that the semantic risk module can be executed without an external cloud service, and the caching mechanism limits the additional inference time introduced by LLM-based risk abstraction. These implementation results address computational feasibility only; they do not constitute deployment validation on an aero-engine test rig or operational fleet. Because the present evaluation is based on C-MAPSS, the observed stopping behavior should be interpreted as benchmark evidence of the proposed decision logic. Validation with experimentally acquired or in-service engine data is required before claims of deployment-level reliability are made. The same local configuration is compatible with air-gapped environments where cloud connectivity is unavailable or prohibited by data governance constraints. On the evaluated hardware, the retrieval of a cached or temporally reused risk level requires a median of 0.08 ms, whereas a fresh local LLM query requires 842.6 ms at the median and 1487.2 ms at the 95th percentile. Because fresh queries occur in only 3.1% of decision cycles, the representative amortized latency of the complete decision path is approximately 245.0 ms. The modular training pipeline—SSAE pre-training on historical fleet data, PPO fine-tuning per engine type—aligns with standard fleet-level prognostics operational practices.

## 6. Conclusions

This paper presented a unified risk-aware predictive maintenance framework for turbofan engines in which prognostic uncertainty is no longer treated as an auxiliary diagnostic quantity but is explicitly integrated into the maintenance decision process. By coupling a probabilistic SSAE predictor, MC Dropout-based uncertainty estimation, CVaR-driven tail-risk quantification, an LLM-based semantic risk layer, and a PPO maintenance policy within a single closed-loop architecture, the proposed RAPM framework establishes a complete degradation-to-decision pipeline that is decision-oriented, interpretable, and methodologically coherent within the evaluated benchmark setting.

The results show that the value of the framework does not lie in maximizing pointwise prediction accuracy alone. Instead, its main contribution is to transform uncertainty into an actionable decision variable. The probabilistic SSAE provides a stable degradation representation with controlled conservatism; the CVaR module converts predictive dispersion into a lower-tail risk signal that improves timing robustness; and the LLM risk layer further lifts numerical risk into a semantically structured representation that reshapes the policy’s notion of urgency. When these components are integrated into the reinforcement learning loop, the resulting policy produces later interventions while maintaining zero failures in the evaluated FD001 episodes, higher optimal stopping rates, and improved robustness to the controlled perturbations considered in the benchmark. Within the matched Rule-PPO control, CVaR yields a 9.0-percentage-point optimal-stopping gain under sensor noise (95% paired interval: [+4.8,+13.4]) and reduces the frequency of terminal RUL below five cycles from 5.8% to 1.4%, linking timing robustness to an improved safety margin.

The independent comparisons reinforce this decision-level advantage: RAPM achieves a 96.0% optimal stopping rate compared with 90.2% for the strongest external maintenance strategy, while controlled substitution with LSTM-MCD, Transformer-MCD, CNN-MCD, and Deep Ensemble backbones retains optimal stopping rates between 92.4% and 95.2% with no late interventions or failures. The direct replacement of the closed-form Gaussian CVaR by the empirical lower-tail average changes the optimal stopping rate by only 0.2 percentage points and preserves zero late interventions and zero failures. After validation-derived bias correction, CVaR Rule-PPO retains a 2.4-percentage-point advantage over its calibrated mean-only counterpart. Matched retraining across 20-, 30-, and 40-cycle planning horizons preserves 94.0–96.0% optimal stopping, whereas the removal of curriculum learning reduces the rate to 58.0% and introduces late interventions and failures. These results establish that the proposed risk-aware decision architecture remains effective across distinct prognostic models, lower-tail estimators, predictive-mean calibrations, and maintenance horizons with curriculum learning providing stable long-horizon policy optimization.

From a broader perspective, this work argues that predictive maintenance in safety-critical systems should not be framed as a point-estimation problem followed by a separate control stage. What ultimately matters is not only how accurately a model predicts the remaining life but whether it can support timely, reliable, and interpretable intervention under uncertainty. The proposed framework addresses this requirement by linking prediction, uncertainty, risk abstraction, and decision optimization in a principled manner. In this sense, the present study is not merely an incremental combination of existing modules but a shift toward decision-centric predictive maintenance.

Several limitations remain. The current study is evaluated only on the model-generated C-MAPSS FD001 benchmark, which represents one operating condition and a simplified HPC degradation scenario. The results therefore provide methodological evidence under a controlled and reproducible simulation benchmark—not direct experimental evidence from a physical aero-engine rig or operational fleet. Validation on experimentally acquired data, additional fault modes, and heterogeneous operating conditions is required before practical deployment claims can be established. Future work should also examine more deterministic semantic risk models, improved uncertainty calibration [51,52], alternative risk-sensitive objectives [29,30], and multi-objective formulations balancing safety, utilization, and maintenance cost. Within this stated scope, the results support the feasibility of using uncertainty-aware and semantically guided risk information to improve maintenance decision timing on a standard aero-engine prognostics benchmark. RAPM therefore provides a reproducible basis for future studies that incorporate physical test-rig or in-service engine data to assess generalization, reliability, and practical deployment.

## Figures and Tables

**Table 1:** Validation sensitivity to the SSAE latent dimension at N=50. Values are validation means from the five-fold protocol with five training seeds. Reconstruction MSE is measured on the normalized sensor scale; RUL MAE is in cycles.

Latent Dimension *d*	Reconstruction MSE	RUL MAE (Cycles)
16	0.041	20.8
32	0.032	19.9
64	0.029	20.3

**Table 2:** MC Dropout sample-size sensitivity for the d=32 SSAE checkpoint.

*N*	Predictive Mean (Cycles)	Total Predictive Std. (Cycles)	Pre-Projection CVaR (Cycles)	95% PICP (%)	Gaussian-Equivalent 95% PI Width (Cycles)
20	78.4±0.3	17.8±0.4	41.7±0.6	91.8±1.2	69.8
50	78.5±0.2	18.1±0.3	41.2±0.4	92.6±1.0	71.0
100	78.5±0.1	18.2±0.2	41.0±0.2	92.8±0.9	71.3

*Note: PICP is computed from the empirical 2.5th and 97.5th percentiles of ${\tilde{y}}_{t}^{\left(n\right)}=\mu_{t}^{\left(n\right)}+\sigma_{t}^{\left(n\right)}\epsilon_{t}^{\left(n\right)}$, so both epistemic and aleatoric uncertainty enter the interval. The final column reports the Gaussian-equivalent central 95% width, $2z_{0.975}\hat{\sigma }=3.9199\hat{\sigma }$, as a common dispersion-scale reference and is not used to compute PICP. Pre-projection CVaR is evaluated cycle-wise as ${\hat{\mu }}_{t}-2.0627{\hat{\sigma }}_{t}$ before aggregation. The ± values denote standard deviations across five random seeds at the seed-mean level.*

**Table 3:** Operational meaning of the semantic risk levels.

Level	Label	Interpretation of the Prognostic Evidence	$\delta_{r}$ (*l_t_*)
1	Safe	The mean and lower-tail RUL indicate substantial remaining life with no immediate maintenance urgency.	0
2	Monitor	Degradation or uncertainty is detectable, but the conservative RUL remains outside the maintenance-critical regime.	0
3	Attention	The prognostic state is approaching a maintenance-relevant regime, or the dispersion gap indicates that closer monitoring and maintenance preparation are warranted.	0
4	Risky	The joint RUL and uncertainty evidence indicates high late-life urgency; continued operation receives an explicit semantic penalty.	−2
5	Critical	The lower-tail estimate indicates imminent end-of-life risk or severe uncertainty in the late-life regime; continued operation receives the strongest semantic penalty.	−5

**Table 5:** Comparison of maintenance decision performance.

Method	Prognostic Backbone	Decision Strategy	Optimal (%)	Early (%)	Late (%)	Failure (%)	RUL at Stop
LSTM-Threshold	LSTM	Fixed threshold	72.6±4.0	21.4±3.6	4.2±1.8	1.8±1.2	31.6±2.7
CNN-MCD-DRL [31]	CNN-MCD	DRL	88.4±3.2	9.6±2.9	2.0±1.3	0.0±0.0	26.8±1.9
BC-SRLA [33]	IOHMM	Hierarchical DRL	85.2±3.6	12.8±3.2	2.0±1.4	0.0±0.0	29.1±2.2
Ensemble-DTS [34]	LightGBM/CatBoost/GB	Dynamic scheduling	90.2±2.7	8.0±2.4	1.8±1.2	0.0±0.0	25.4±1.8
RAPM	Probabilistic SSAE	CVaR-LLM-PPO	96.0±2.1	4.0±2.1	0.0±0.0	0.0±0.0	23.2±1.4

**Table 6:** Controlled effect of prognostic-backbone substitution on maintenance decisions.

Prognostic Backbone	Decision Layer	Optimal (%)	Early (%)	Late (%)	Failure (%)	RUL at Stop
LSTM-MCD	CVaR + LLM-PPO	93.2±2.6	6.8±2.6	0.0±0.0	0.0±0.0	24.6±1.6
Transformer-MCD	CVaR + LLM-PPO	95.2±2.2	4.8±2.2	0.0±0.0	0.0±0.0	23.8±1.5
CNN-MCD	CVaR + LLM-PPO	92.4±2.8	7.6±2.8	0.0±0.0	0.0±0.0	25.1±1.7
Deep Ensemble	CVaR + LLM-PPO	94.6±2.3	5.4±2.3	0.0±0.0	0.0±0.0	24.0±1.5
Probabilistic SSAE	CVaR + LLM-PPO	96.0±2.1	4.0±2.1	0.0±0.0	0.0±0.0	23.2±1.4

**Table 7:** Gaussian-CVaR approximation and decision sensitivity.

**(a) Distributional Diagnostics and Lower-Tail Estimation Error**							
**Region**	**Median Skewness**	**Excess Kurtosis**	**AD Rejection (%)**	**CVaR Bias (Cycles)**		**CVaR MAE (Cycles)**	**P95 $|e_{t}^{\mathrm{CVaR}}|$ (Cycles)**
All cycles	0.42	0.85	28.0	+0.6		1.8	4.1
RUL≤30	0.91	1.72	46.0	+1.1		2.4	4.8
**(b) Frozen-Policy Estimator Substitution**							
**CVaR Estimator**	**Optimal (%)**	**Early (%)**	**Late (%)**	**Failure (%)**	**RUL at Stop**	**Risk-Level Disagreement (%)**	**Action Disagreement (%)**
Gaussian closed form	96.0±2.1	4.0±2.1	0.0±0.0	0.0±0.0	23.2±1.4	–	–
Empirical (N=50)	95.8±2.2	4.2±2.2	0.0±0.0	0.0±0.0	23.3±1.5	4.8	1.6

**Table 8:** Bias-correction control experiment.

**(a) RUL Prediction After Validation-Derived Calibration**							
**Prediction**	**Bias (Cycles)**	**MAE**	**RMSE**	**MAE for RUL≤30**		**95% PICP (%)**	
Original SSAE	−16.7	19.9	24.1	13.1		92.6	
Bias-corrected SSAE	+0.2	13.9	17.4	12.3		93.8	
**(b) Matched 2×2 Rule-PPO Decision Control**							
**Mean Calibration**	**Decision Variant**	**Optimal (%)**	**Early (%)**	**Late (%)**	**Failure (%)**	**RUL at Stop**	**Avg Reward**
Original	No-CVaR PPO	92.0±2.3	8.0±2.3	0.0±0.0	0.0±0.0	24.1±1.5	327.8±13.1
Original	CVaR Rule-PPO	95.0±1.8	5.0±1.8	0.0±0.0	0.0±0.0	23.2±1.4	333.8±12.5
Bias-corrected	No-CVaR PPO	93.4±2.2	6.6±2.2	0.0±0.0	0.0±0.0	23.8±1.5	330.2±12.8
Bias-corrected	CVaR Rule-PPO	95.8±1.9	4.2±1.9	0.0±0.0	0.0±0.0	23.3±1.4	334.5±12.2

**Table 9:** Policy performance under different perturbations.

Variant	Perturbation	Optimal (%)	Early (%)	Late (%)	Failure (%)	Avg RUL at Stop	Avg Reward
Rule-PPO	Baseline	95.0±1.8	5.0±1.8	0.0±0.0	0.0±0.0	23.2±1.4	333.8±12.5
Rule-PPO	Data noise, σ=0.05	82.0±2.6	18.0±2.6	0.0±0.0	0.0±0.0	26.3±1.8	303.7±15.2
Rule-PPO	Latent-feature perturbation, σ=0.10	96.0±1.7	4.0±1.7	0.0±0.0	0.0±0.0	23.1±1.3	336.2±11.9
No-CVaR PPO	Baseline	92.0±2.3	8.0±2.3	0.0±0.0	0.0±0.0	24.1±1.5	327.8±13.1
No-CVaR PPO	Data noise, σ=0.05	73.0±3.1	27.0±3.1	0.0±0.0	0.0±0.0	27.8±2.1	284.2±17.4
No-CVaR PPO	Latent-feature perturbation, σ=0.10	92.0±2.4	8.0±2.4	0.0±0.0	0.0±0.0	24.1±1.6	328.0±13.3
Random Policy	Baseline	0.0±0.0	100.0±0.0	0.0±0.0	0.0±0.0	123.8±4.5	−159.6±8.2

**Table 10:** Paired inference and terminal safety margins.

Condition	$\Delta_{\mathrm{opt}}$ (pp)	Paired Bootstrap 95% CI (pp)	CVaR $q_{<5}$ (%)	No-CVaR $q_{<5}$ (%)
Baseline	+3.0	[−0.6,+6.8]	0.6	2.2
Data noise	+9.0	[+4.8,+13.4]	1.4	5.8
Latent perturbation	+4.0	[+0.4,+7.8]	0.8	2.8

**Table 11:** Stopping-window sensitivity.

Protocol	*W*	Optimal (%)	Early (%)	Late (%)	Failure (%)	RUL at Stop (Cycles)
Fixed W=30 policy	20	30.0±2.1	70.0±2.1	0.0±0.0	0.0±0.0	23.2±1.4
Fixed W=30 policy	30	96.0±2.1	4.0±2.1	0.0±0.0	0.0±0.0	23.2±1.4
Fixed W=30 policy	40	99.0±0.9	1.0±0.9	0.0±0.0	0.0±0.0	23.2±1.4
Matched retraining	20	94.0±2.3	6.0±2.3	0.0±0.0	0.0±0.0	16.5±1.2
Matched retraining	30	96.0±2.1	4.0±2.1	0.0±0.0	0.0±0.0	23.2±1.4
Matched retraining	40	95.2±2.0	4.8±2.0	0.0±0.0	0.0±0.0	30.8±1.6

**Table 12:** Curriculum-learning ablation.

Training Scheme	Start Fractions	Step Boundaries	Optimal (%)	Early (%)	Late (%)	Failure (%)
Three-phase curriculum	0.88/0.70/0.00	30k/80k/150k	96.0±2.1	4.0±2.1	0.0±0.0	0.0±0.0
Naive full-lifecycle PPO	0.00 throughout	150k	58.0±5.2	32.0±4.8	6.0±2.1	4.0±1.8

**Table 13:** Paired risk-level comparison on Engine 99.

Relation on Shared Decision Steps	Number of Steps	Proportion (%)
LLM level higher than rule level	118	88.1
Equal levels	6	4.5
LLM level lower than rule level	10	7.5
Total	134	100.0

The shared interval includes both endpoint indices. Individual percentages are rounded to one decimal place.

**Table 14:** LLM calls per episode under different scheduling configurations.

Configuration	Calls per Episode	Total Calls (500 Episodes)	Reduction vs. No Mechanism
No throttling, no caching	183.1±26.4	91,550	–
Throttling only (K=25)	7.9±1.1	3950	95.7%
Semantic caching only	22.4±4.2	11,200	87.8%
Throttling + caching	5.6±0.8	2800	96.9%

*Note: The semantic cache is initialized empty at the start of every episode and is never shared across engine trajectories, folds, or seeds. Consequently, the first unseen cache key in each episode requires a fresh evaluation.*

**Table 15:** Measured component latency of RAPM.

Operation	Execution Frequency	Median Latency (ms)	P95 Latency (ms)
Single SSAE forward pass (reference)	Profiling only	4.8	6.2
Probabilistic SSAE (N=50)	Every decision cycle	218.5	276.3
CVaR, dispersion-gap, and state construction	Every decision cycle	0.12	0.18
PPO action selection	Every decision cycle	0.21	0.29
Cached or temporally reused risk level	Non-query cycles	0.08	0.12
Fresh Qwen2.5-3B risk query	$p_{q}$ of decision cycles	842.6	1487.2

## Data Availability

The C-MAPSS benchmark is publicly available from NASA [45].

## Appendix A. Reference Specification of the LLM Risk Interface

### Appendix A.1. Prompt and Inference Configuration

The reference interface maps the four-component context $c_{t}=\left(t,{\hat{\mu }}_{t},{\mathrm{CVaR}}_{t},\Delta_{t}^{\mathrm{unc}}\right)$ to an ordinal risk level. Each fresh request contains one system message and one user message with no conversation history. The input excludes reference RUL, the failure cycle, future observations, and maintenance outcomes. The interface accepts the unrounded numerical state; the user-message fields are serialized to three decimal places, while cache keys and fallback decisions use the full-precision values.

The reference deployment uses the instruction-tuned Qwen2.5-3B model [15], which was served as qwen2.5:3b-instruct-q4_K_M.

Requests are sent to Ollama’s /api/chat endpoint and specify format="json" and stream=false with the following explicit decoding options:

{"temperature": 0.1, "num_predict": 120, "num_ctx": 2048,

 "top_k": 40, "top_p": 0.9, "min_p": 0.0,

 "repeat_penalty": 1.0, "seed": 0}

The decoding seed belongs to the reference risk interface and is distinct from the seeds used to train the prognostic and PPO models. Each request has a 30 s timeout and is attempted once. The system message is shown below:

You are a reliability risk assessor for turbofan maintenance.

Assess only the numerical evidence in the user message.

All remaining-life quantities are measured in operating cycles.

mean_rul is the predictive mean. cvar_rul is the nonnegative,

physically projected lower-tail RUL at alpha=0.05.

dispersion_gap is 2.063 times the total predictive standard

deviation, before CVaR clipping. It measures dispersion, not bias.

cycle is elapsed time; it does not reveal the failure horizon.

Assess mean RUL, lower-tail RUL, and dispersion jointly.

Use 30 cycles as the nominal maintenance-planning reference.

For otherwise comparable states, lower mean or lower-tail RUL

indicates greater urgency. Greater dispersion strengthens

urgency, particularly when remaining life is already short.

A nonpositive mean or near-zero lower tail warrants critical

attention. Elapsed time alone does not establish degradation.

Do not infer true RUL, future measurements, or a failure date.

Use these ordinal meanings:

1 Safe: substantial mean and lower-tail remaining life.

2 Monitor: detectable degradation or uncertainty with margin.

3 Attention: approaching the maintenance regime or appreciable

  uncertainty requiring closer monitoring and preparation.

4 Risky: high late-life urgency from the joint evidence.

5 Critical: near-zero lower-tail life or severe late-life risk.

Return exactly one JSON object with only these two fields:

{"risk_level": <integer 1-5>, "reason": "<brief justification>"}

Use one sentence of at most 25 words and 240 characters for reason.

Do not add markdown, extra keys, or a stop/continue command.

The user message uses the following literal template; braces identify fields substituted by the caller:

cycle={cycle:d}

mean_rul={mean_rul:.3f}

cvar_rul={cvar_rul:.3f}

dispersion_gap={dispersion_gap:.3f}

Return the risk JSON object.

The prompt fixes the evidence, ordinal semantics, and output contract. The returned justification is retained for audit, while only the validated integer $l_{t}$ enters the PPO state and continuation reward.

### Appendix A.2. Response Validation

Validation parses the complete response text in message.content. Leading and trailing whitespace is removed, and the remaining string must decode as one JSON object with exactly the keys risk_level and reason. Duplicate keys, non-finite constants, additional text, and fenced code blocks are rejected. The risk level must be a JSON integer in {1,…,5}; Boolean values, decimal numbers, numeric strings, and out-of-range values are rejected without coercion or clipping. The justification must be a nonempty string containing at most 25 whitespace-delimited words and 240 characters after trimming. The following Python 3.13 routine defines the acceptance rule:

import json

def _reject_constant(value):

    raise ValueError(“Non-finite JSON constant”)

def _unique_object(pairs):

    result = {}

    for key, value in pairs:

        if key in result:

            raise ValueError(“Duplicate JSON key”)

        result[key] = value

    return result

def parse_risk(text):

    if not isinstance(text, str):

        raise ValueError(“Missing response text”)

    obj = json.loads(text.strip(), object_pairs_hook=_unique_object,

                     parse_constant=_reject_constant)

    if type(obj) is not dict or set(obj) != {“risk_level”, “reason”}:

        raise ValueError(“Invalid response fields”)

    level, reason = obj[“risk_level”], obj[“reason”]

    if type(level) is not int or not 1 <= level <= 5:

        raise ValueError(“Invalid risk level”)

    if not isinstance(reason, str):

        raise ValueError(“Invalid justification”)

    reason = reason.strip()

    if not reason or len(reason) > 240 or len(reason.split()) > 25:

        raise ValueError(“Invalid justification length”)

    return level, reason

An HTTP failure, timeout, incomplete response, invalid response envelope, or validation exception invokes the deterministic mapping below on the same numerical context. The response is not repaired by extracting an arbitrary digit, and no second language-model query is issued.

### Appendix A.3. Deterministic Fallback

The reference RuleRisk mapping orders the projected lower-tail RUL and adds one level of urgency for pronounced dispersion in the maintenance-relevant regime. With $c={\mathrm{CVaR}}_{t}$, its base level is

$$b\left(c\right)=\left\{\begin{array}{cc} 1, & c>90,\\ 2, & 60<c\leq 90,\\ 3, & 30<c\leq 60,\\ 4, & 10<c\leq 30,\\ 5, & 0\leq c\leq 10. \end{array}\right.$$

The dispersion adjustment and final level are

$$u_{t}=1\;\left({\hat{\mu }}_{t}\leq 60\;\wedge \;\Delta_{t}^{\mathrm{unc}}\geq 10\right),\;l_{t}^{\mathrm{rule}}=\left\{\begin{array}{cc} 5, & {\hat{\mu }}_{t}\leq 0,\\ \min\{5,b\left({\mathrm{CVaR}}_{t}\right)+u_{t}\}, & {\hat{\mu }}_{t}>0. \end{array}\right.$$

All thresholds are in cycles. The 10-, 30-, 60-, and 90-cycle boundaries define nested urgency regions around the nominal 30-cycle planning horizon. They remain fixed when the reward horizon *W* is varied. For fixed predictive location, a smaller lower-tail estimate or a larger dispersion gap cannot reduce the fallback level. The substitution of ${\mathrm{CVaR}}_{t}={\hat{\mu }}_{t}$ and $\Delta_{t}^{\mathrm{unc}}=0$ gives the corresponding mean-only rule for the No-CVaR control. This mapping also defines the deterministic reference alternative to the language-model assessment.

### Appendix A.4. Cache and Temporal Reuse

The reference scheduler follows Algorithm 1. It first looks up the full-precision, five-cycle-bin key in Equation (8). A hit supplies the stored level immediately. On a miss, a fresh query is issued only when at least 25 decision steps have elapsed since the previous attempt; otherwise, the latest level is reused. At an episode start $t_{0}$, the cache is cleared, the last-attempt index is initialized to $t_{0}-25$ and the previous level to 1, so the first cache miss is eligible for inference. Both a validated LLM result and a fallback result are stored under the current key and advance the last-attempt index. Cache hits do not advance that index. The previous level is updated after every decision step, including a cache hit.

Cache entries are confined to one episode and are never transferred between engines, folds, seeds, or training, validation, and held-out evaluation. Cycles are processed in temporal order. Changes to the prognostic checkpoint, calibration, prompt, or decoding options initialized a new cache. Each decision record retains the numerical context, cache key, final level, and its origin (fresh inference, cached inference, temporal reuse, or fallback). Fresh-query records additionally retain the raw response and validation status. The accompanying risk_advisor_reference.py implements this interface and its deterministic fallback.
